# AIG1 affects in vitro and in vivo virulence in clinical isolates of *Entamoeba histolytica*

**DOI:** 10.1371/journal.ppat.1006882

**Published:** 2018-03-19

**Authors:** Kumiko Nakada-Tsukui, Tsuyoshi Sekizuka, Emi Sato-Ebine, Aleyla Escueta-de Cadiz, Dar-der Ji, Kentaro Tomii, Makoto Kuroda, Tomoyoshi Nozaki

**Affiliations:** 1 Department of Parasitology, National Institute of Infectious Diseases, Tokyo, Japan; 2 Laboratory of Bacterial Genomics, Pathogen Genomics Center, National Institute of Infectious Diseases, Tokyo, Japan; 3 Center for Research and Diagnostics, Centers for Disease Control, Taipei, Taiwan; 4 Artificial Intelligence Research Center (AIRC) and Biotechnology Research Institute for Drug Discovery, National Institute of Advanced Industrial Science and Technology (AIST), Tokyo, Japan; 5 Graduate School of Life and Environmental Sciences, University of Tsukuba, Ibaraki, Japan; 6 Department of Biomedical Chemistry, Graduate School of Medicine, The University of Tokyo, Tokyo, Japan; University of Virginia Health System, UNITED STATES

## Abstract

The disease state of amebiasis, caused by *Entamoeba histolytica*, varies from asymptomatic to severe manifestations that include dysentery and extraintestinal abscesses. The virulence factors of the pathogen, and host defense mechanisms, contribute to the outcomes of infection; however, the underlying genetic factors, which affect clinical outcomes, remain to be fully elucidated. To identify these genetic factors in *E*. *histolytica*, we used Illumina next-generation sequencing to conduct a comparative genomic analysis of two clinical isolates obtained from diarrheal and asymptomatic patients (strains KU50 and KU27, respectively). By mapping KU50 and KU27 reads to the genome of a reference HM-1:IMSS strain, we identified two genes (EHI_089440 and EHI_176590) that were absent in strain KU27. In KU27, a single *AIG1* (*avrRpt2*-induced gene 1) family gene (EHI_176590) was found to be deleted, from a tandem array of three *AIG1* genes, by homologous recombination between the two flanking genes. Overexpression of the EHI_176590 gene, in strain HM-1:IMSS cl6, resulted in increased formation of cell-surface protrusions and enhanced adhesion to human erythrocytes. The EHI_176590 gene was detected by PCR in 56% of stool samples from symptomatic patients infected with *E*. *histolytica*, but only in 15% of stool samples from asymptomatic individuals. This suggests that the presence of the EHI_176590 gene is correlated with the outcomes of infection. Taken together, these data strongly indicate that the AIG1 family protein plays a pivotal role in *E*. *histolytica* virulence via regulation of host cell adhesion. Our *in-vivo* experiments, using a hamster liver abscess model, showed that overexpression or gene silencing of EHI_176590 reduced and increased liver abscess formation, respectively. This suggests that the *AIG1* genes may have contrasting roles in virulence depending on the genetic background of the parasite and host environment.

## Author summary

Comparative genomic analysis of pathogens is used to identify genes associated with disease manifestations. In the present study, this approach was used to identify genes, present in a strain of *Entamoeba histolytica* isolated from dysenteric patients, but absent in strains isolated from asymptomatic infected individuals. Strain KU27 is an “avirulent” strain, or less virulent as compared to the virulence of other strains. KU27 was isolated from an asymptomatic infected individual, and is incapable of producing abscesses in a hamster liver abscess model. A previous genotyping study, using tRNA-linked short tandem repeats as markers, also identified this Japanese strain as a genetically distinct low-virulence strain. We conducted a comparative genomic analysis of strain KU27 against virulent strain of *E*. *histolytica*. Our analysis identified an *AIG1* gene as a potential determinant of virulence. Further, we demonstrated that the gene product is involved in the formation of protrusions on the plasma membrane and adherence to host cells. To the best of our knowledge, this is the first study to identify a virulence-associated gene by comparative genomics. Here, we demonstrate the causal connection of the identified gene with virulence by the analysis of cell morphology, adhesion competence, and distribution among clinical isolates.

## Introduction

Amebiasis, a common protozoan infection in developing and developed countries, results in an estimated 40,600 to 73,800 deaths annually [1]. Amebiasis occurs upon ingestion of water or food contaminated with dormant cysts of *Entamoeba histolytica*; the cysts, then, form motile trophozoites that colonize the colon. Almost 90% of individuals, infected with *E*. *histolytica*, show no symptoms or only subclinical manifestations [2]. Symptomatic cases often involve colitis and dysentery, with 5–10% of symptomatic individuals developing extraintestinal invasive amebiasis; this may lead to the formation of amoebic liver abscesses (ALA) [3].

The factors that determine the outcome of amebiasis are not well understood, although host defense mechanisms and pathogen virulence are presumed to be the major contributing factors. Cohort studies in Bangladesh revealed that polymorphisms in several genes, including those encoding human leukocyte antigen (HLA) and the leptin receptor, are involved in susceptibility to infection with *E*. *histolytica* (reviewed in [4]). Specifically, Bangladeshi children who harbor the DQB1*0601 heterozygous and homozygous haplotypes of HLA, and those with a Q223R substitution in the leptin receptor, are more susceptible to infection with *E*. *histolytica* [5, 6]. In regard to parasitic factors, several virulence factors have been characterized to date [7–9]; however, no single determinant, conferring virulence, has been identified. Several studies conducted transcriptomic comparisons between *E*. *histolytica* strains that differ in virulence or between *E*. *histolytica* and the non-virulent *E*. *dispar* [10–13].

Detecting the genome/genes associated with the outcomes of infection is mainly based on genotyping repeat-containing genes, such as chitinase and serine-rich *E*. *histolytica* protein (SREHP) [14–16], and tRNA-linked short tandem repeats in the non-coding regions [17–19]. In the latter case, genotype 5RR, in the R-R locus, is associated with asymptomatic cases, while genotype 10RR is associated with symptomatic cases [19]. However, the genotyping of *E*. *histolytica* isolates has not resulted in the unequivocal identification of the gene(s) responsible for pathogenesis and infection outcomes, such as symptom development and severity, and organ tropism. In a study of genome comparisons, Weedall and colleagues attempted to identify genetic differences between high and low virulence isolates of *E*. *histolytica* [20]. Their study found putative single-nucleotide polymorphisms among the isolates, in genes and gene families with putative roles in virulence. They also found differences in gene copy numbers between genomes, indicating that recombination has occurred. However, they were unable to identify genes or single-nucleotide polymorphisms that correlated with either geographic and temporal origins, or disease outcomes. The reasons behind this apparent lack of association were not clear, but it could be due to the broad range of geographical regions (Mexico, Venezuela, Bangladesh, Korea, United Kingdom, and Italy) and extended period of time (1951–2007) required to obtain the isolates used in the study. To overcome these potential problems, we used recent (i.e., temporally restricted) Japanese (i.e., geographically restricted) clinical strains for an independent analysis, aiming to identify the *E*. *histolytica* gene(s) that strongly influence infection outcomes. We conducted comparative genomic analyses of two representative clinical *E*. *histolytica* strains, KU50 and KU27, which were isolated from dysenteric and asymptomatic patients, respectively, in Japan, [15, 16, 21]. Strain KU27 was previously shown to be incapable of producing ALA in an animal model, whereas KU50 retains that ability [21]. Our analyses revealed that the virulent strain contains a gene in a single genetic locus that is missing in the low-virulence KU27 strain. This gene was found to encode an AIG1 family protein involved in the formation of cell protrusions and adhesion to the host cells.

## Results

### Comparative genomic analysis of KU27 and KU50 and identification of gene deletion

Short genomic reads of low-virulence KU27 and high-virulence KU50 strains were sequenced using an Illumina GAIIx sequencer and mapped against the HM-1:IMSS (HM-1) reference genome (GenBank WGS_SCAFLD NW_001914860-NW_001916388). The percentage of mapped reads was approximately 80 and 72% for strains KU27 and KU50, respectively, and the median coverage was approximately 170 fold (S1 Table). *De novo* assembly of the obtained reads from KU27 and KU50 will be described elsewhere. Briefly, the total contig size, number of contigs, N50, and maximal contig size were approximately 26 Mb, 8,500, 5 kb, and 60 kb, respectively. To validate the close relationship between the virulent and less-virulent strains, we conducted the core genome phylogenetic and single nucleotide variation (SNV) analyses of strains KU27 and KU50, as well as other *E*. *histolytica* strains including HM-1: IMSS, HM-1:IMSS-A, HM-1:IMSS-B, HM-3:IMSS, DS4-868, MS96-3382, and Rahman (S7A Fig). In previous studies, strains Rahman and HM-1:IMSS were used as representative low-virulence and high-virulence strains, respectively, as assessed by comparative genomic and transcriptomic analyses [20, 22, 23]. The number of pairwise SNVs, between KU27 and KU50, showed lower variation than those between Rahman and HM-1:IMSS, 2436 and 3259, respectively (S7A Fig).

**Fig 1:**
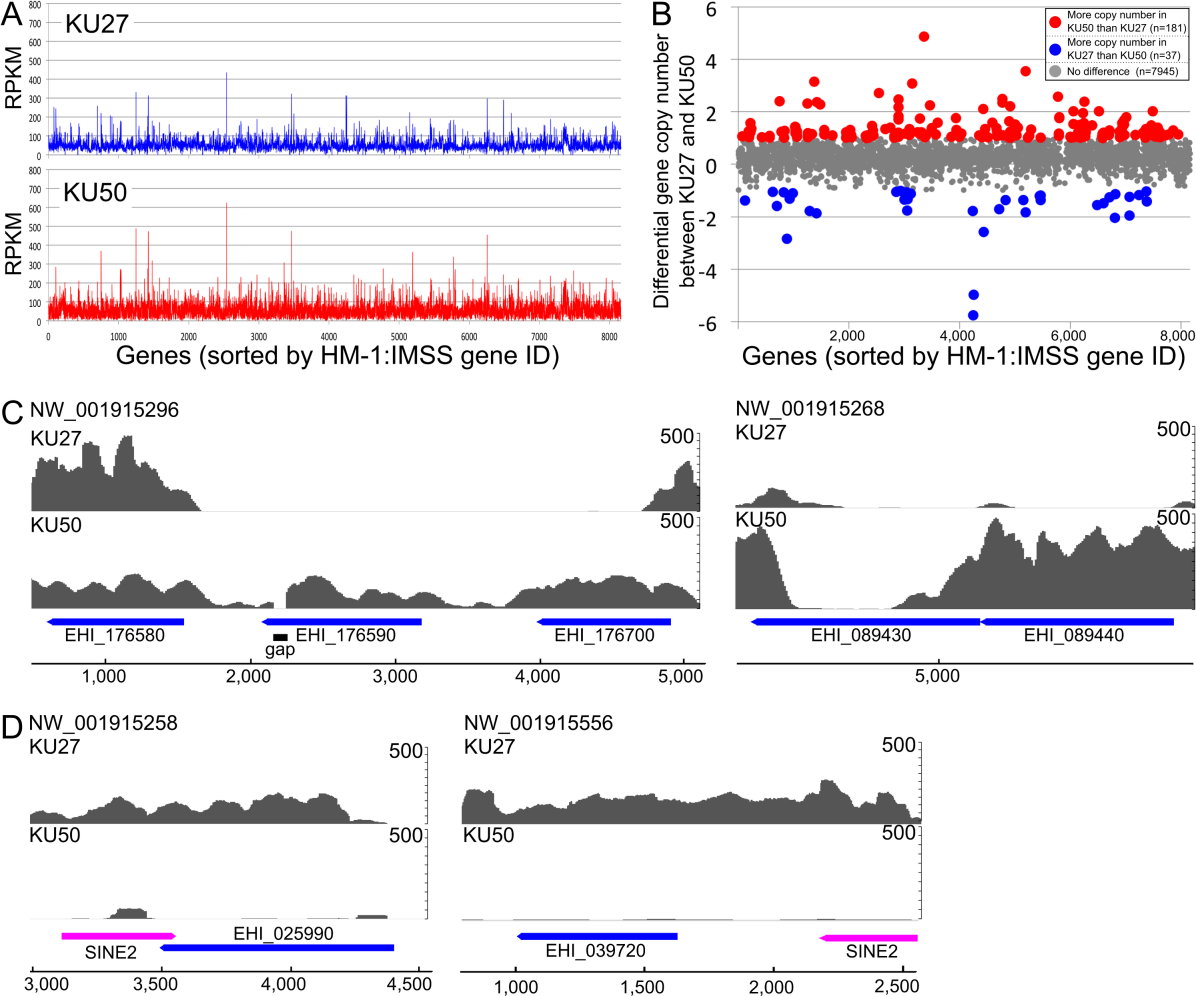
Comparative genome analysis of *E*. *histolytica* strains KU27 and KU50. **(**A) RPKM score of the genomes of strains KU27 and KU50. The X-axis represents 8,163 annotated genes of *E*. *histolytica* HM-1:IMSS in the order of locus tag gene ID. (B) Gene copy number variations in KU27 and KU50. RPKM scores of 3252 genes, with a higher value than the RPKM median of all genes, are shown. The 181 genes that are present at higher copy number in KU50, compared to that in KU27, are displayed as red dots; the 37 genes, present in higher copy number in KU27 compared to that in KU50, are shown as blue dots. The x-axis represents 8,163 annotated *E*. *histolytica* HM-1:IMSS genes ordered by locus tag gene ID. The y-axis represents the number of gene copies, per gene, expressed as KU50 gene copy number minus KU27 gene copy number. (C, D) Raw mapping data of KU27 and KU50 reads to the regions that contain genes present in KU50, but absent in KU27 (C), and those present in KU27, but absent in KU50 (D). *E*. *histolytica* HM-1:IMSS reference genome sequence was used for mapping. The y-axis displays mapping read coverage.

We observed gene copy number variation between the two strains. Gene copy variations were also previously described by Weedall et al. [20] (Fig 1A). The mapping and reads per kilobase of exon per million mapped reads (RPKM) data (Fig 1A) indicated various biases in the coverage depth of genes between KU27 and KU50; this suggests the existence of variations in gene copy number (Fig 1B). Among 8163 genes, 218 genes showed differences of >1 copy number between KU27 and KU50 (Fig 1B, shown as red and blue dots). Moreover, four genes (EHI_025990, EHI_039720, EHI_089440, and EHI_176590) were predicted to be absent in one of the strains (KU27 or KU50; as indicated by the “zero” in S2 Table). A volcano plot, based on the RPKM value of each ORF between strains KU27 and KU50, also indicated differential copy numbers of these genes (S7B Fig). Raw read mapping data verified the absence of these genes in the corresponding strain (Fig 1C and 1D). EHI_089440 and EHI_176590 are present in strains HM-1 and KU50, but are absent in strain KU27 (Fig 1C). An approximately 4-kb region, which contains EHI_176590 and parts of the upstream and downstream genes, was missing only in KU27 (Fig 1C, S2 Table); this finding was further verified by sequencing analysis (see below). Comparison of the mapping and RPKM data also revealed that EHI_025990 and EHI_039720 were missing in strain KU50 (Fig 1D).

We further compared the RPKM values of the four ORFs, missing in KU27, with those of the 10 *E*. *histolytica* strains analyzed by Weedall and colleagues [20] (S5 Table). Genes EHI_176590 and EHI_089440, deleted in the low-virulence KU27 strain, were absent or showed a low RPKM value (0.7–3.8), respectively, in strains HK-9, PVBM08B, and PVBM08F. The average RPKM values of other seven strains, for EHI_176590 and EHI_089440, were 41.5 and 74.8, respectively. One of the two genes, EHI_025990, deleted in the high-virulence KU50 strain, was absent in strains MS84-1373, Rahman, and IULA:1092:1; EHI_039720, and was not analyzed in this study [20]. Rahman and MS84-1373 were isolated from asymptomatic individuals and the rest of the strains were isolated from patients with colitis/dysentery [20].

### Confirmation of the loss of EHI_176590 in strain KU27

**Fig 2:**
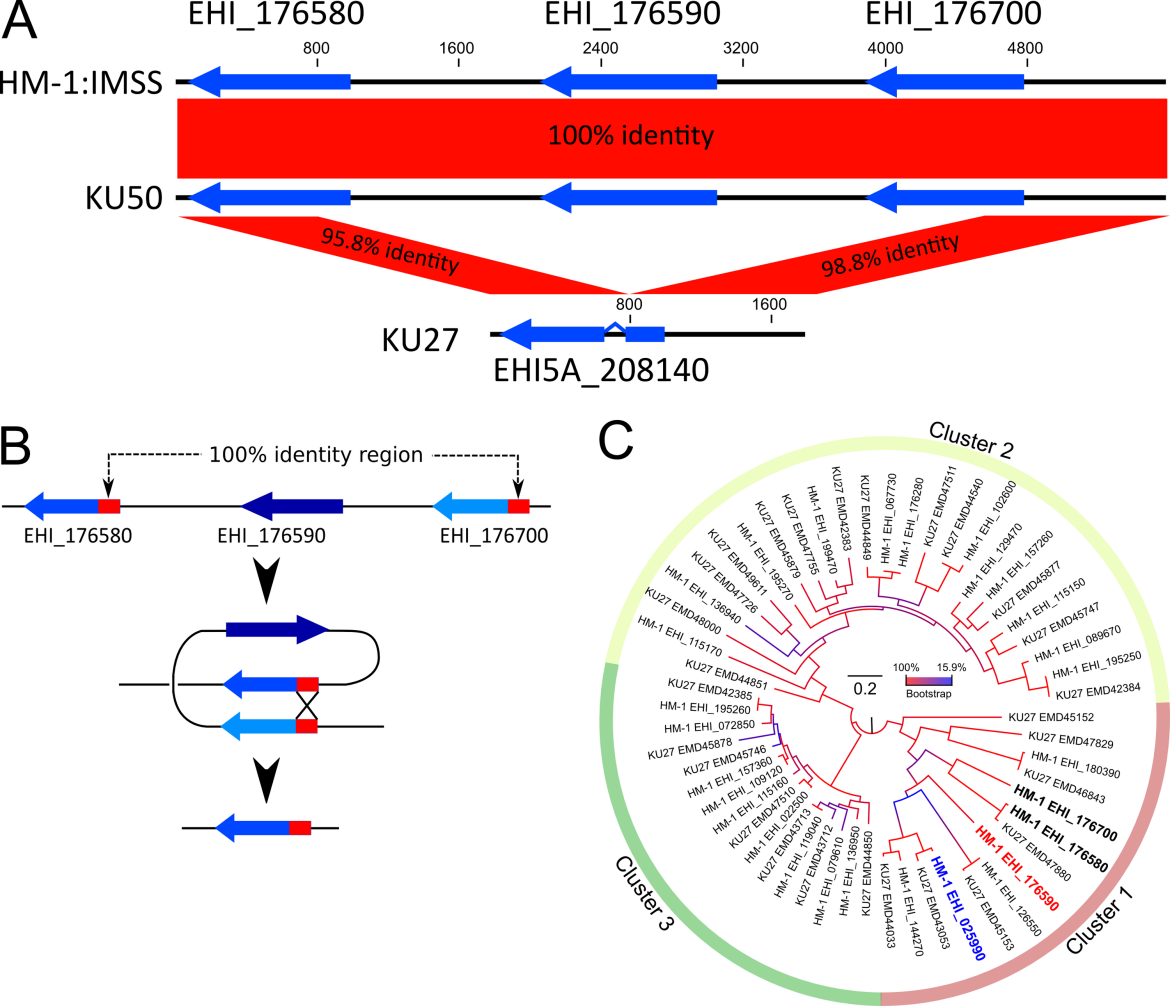
Schematic representation of the AIG1 family protein gene-containing region that is missing in strain KU27, and phylogenetic analysis of AIG1 family proteins. (A) Graphical view of the region, containing AIG1 family protein genes, in strains HM-1, KU50, and KU27. Three AIG1 genes in strains HM-1 and KU50, and one recombinant gene in strain KU27, are depicted with blue arrows. (B) A depiction of a recombination event that likely resulted in loss of the EHI_176590 gene in KU27. (C) Phylogenetic tree of AIG1 family proteins in HM-1 and strain KU27. A best maximum likelihood tree was produced using 28 and 27 AIG1 family protein sequences from strain HM-1 and KU27, respectively. Bootstrap values, at each node, with 1000 replicates, are color coded. The scale bar indicates the number of amino acid substitutions per site.

**Fig 3:**
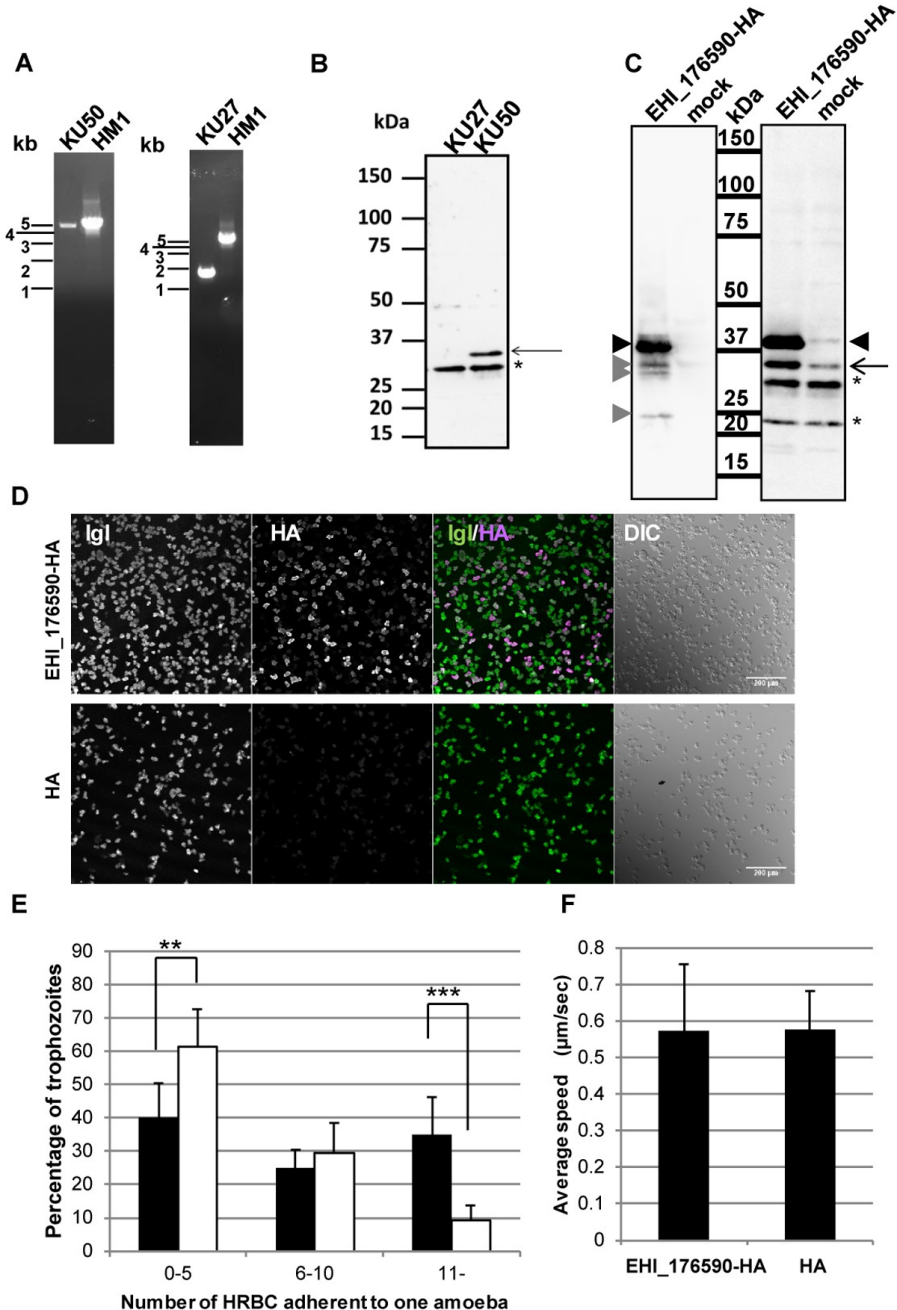
Verification of the loss of EHI_176590 in KU27, overexpression of EHI_176590, and phenotypes caused by EHI_176590 overexpression. (A) Verification by PCR of the lost genomic region, containing the EHI_176590 gene, in KU27. (B) Immuno-detection of EHI_176590 in KU27 and KU50. Total cell lysates, from the indicated strains, were analyzed by immunoblot analysis with anti-EHI_176590 antibody. The arrow depicts the EHI_176590 protein, which is present in KU50, but missing in KU27; asterisk indicates the protein band detected by non-specific binding or cross reaction. (C) Immuno-detection of EHI_176590-HA. Total lysates from EHI_176590-HA-expressing and mock transformants were analyzed by immunoblot analysis with an anti-HA antibody (left panel) or anti-EHI_176590 antibody (right panel). Black arrowheads indicate full-length EHI_176590-HA, and gray arrowheads indicate minor truncated forms of EHI_17690-HA. The arrow indicates intrinsic EHI_176590. Asterisks depict protein bands detected by non-specific binding or cross reaction. (D) Low magnification confocal immunofluorescence images of the EHI_176590-HA-expressing and mock-transfected transformants. Trophozoites were fixed and reacted with anti-Igl and anti-HA antibodies. Merged fluorescent (IGL/HA) and differential interference contrast (DIC) images are also shown. (E) Increased adherence to HRBCs by EHI_176590 overexpression. EHI_176590-HA-expressing (filled bars) and mock (open bars) transformants were co-cultured with HRBCs on ice for 30 min, and the number of adherent HRBCs per ameba were counted. The total number of trophozoites was set to 100% and the percentages of trophozoites, bound to 0–5, 6–10, or >10 HRBCs, are shown. Error bars indicate standard deviations of four biological replicates (2292 and 1762 trophozoites were counted for the EHI_176590-HA-expressing and mock transformants, respectively). **p-value <0.01, ***p-value <0.001 (F) Cell motility of the EHI_176590-HA-expressing and mock-transfected transformants. Time-lapse images of CellTracker Green-loaded EHI_176590-HA-expressing and mock (“HA”) transformants were analyzed by ICY software to measure cell motility. The average speed and standard deviation of the motility of EHI_176590-HA and mock vector transformants (HA) are shown; 135 and 106 trophozoites, respectively, were monitored in four independent experiments.

To verify that the genomic region, containing EHI_176590, is missing in KU27, we analyzed contigs assembled *de novo* and Sanger sequencing data. Because the contig, corresponding to this region in HM-1 (NW_001915296), contains a gap at nucleotide positions 2034–2133 (Fig 1C), the gap was filled by PCR and Sanger sequencing of the encompassing region from HM-1 and KU50. The gap region in HM-1 was 517 bp in length. Comparative analysis of the region and flanking genes showed that the nucleotide sequence was identical between HM-1 and KU50 (Fig 2A). In contrast, a 3,802-bp region was deleted in strain KU27 (Figs 2A and 3A). Furthermore, a 1,011-bp upstream region, containing the EHI_176700 gene, and a 772-bp region containing the downstream EHI_176580 gene, showed 98.8 and 95.8% positional identity, respectively, at the nucleotide level. These data indicate that the EHI_176590 gene and parts of the two flanking genes were lost in strain KU27 (Fig 2A). The EHI_176580 and EHI_176700 genes share high similarity, particularly at the 5’ end of the protein-coding region, which contains a 194-bp sequence that is identical between the two genes; this would allow for homologous recombination (Fig 2B), yielding the truncated region in strain KU27. As a consequence of this truncation, the EHI_175580 gene in KU27 is present as an intron-containing, fused gene composed of EHI_175580 and EHI_175700 (annotated as EHI5A_208140) (Fig 2A).

### Overexpression of AIG1 family protein EHI_176590 causes accumulation of endogenous EHI_176590

Among the four genes, EHI_025990, EHI_039720, EHI_089440, and EHI_176590, which were differentially present in strains KU27 and KU50/HM-1, only the EHI_176590 gene showed significant levels of mRNA expression in KU50, as assessed by a custom-made DNA microarray [24–27] (S1 Fig). The three other genes showed low, negligible levels of expression, in the range of 0.7–1.5% of the expression shown by RNA polymerase II gene (EHI_056690). This finding prompted us to examine the role of the EHI_176590 protein in the virulence of *E*. *histolytica*.

The expression of EHI_176590 protein was examined by immunoblot analysis using an anti-EHI_176590 antibody (Fig 3B). The specificity of the antibody to the 32-kDa EHI_176590 protein was confirmed using a strain in which the expression of the EHI_176590 gene was repressed by gene silencing [28] (S2 Fig). The apparent molecular mass of the EHI_176590 protein was smaller than predicted (36 kDa, 320 amino acids); a similar observation was also made for HA-tagged EHI_176590 protein (EHI_176590-HA), which was determined to have a mass of 37 kDa. The EHI_176590 protein was detected in strain KU50, but not in strain KU27 (Fig 3B).

To investigate the function of the EHI_176590 protein in *E*. *histolytica*, we generated a transgenic ameba line that overexpresses EHI_176590-HA using the standard laboratory strain HM-1:IMSS cl-6. The expression of EHI_176590-HA was confirmed by immunoblot analysis using anti-HA and EHI_176590 antibodies (Fig 3C); the analysis detected EHI_176590-HA as a major 37-kDa band (with several minor truncated proteins) in the transformant. Notably, the EHI_176590-HA-expressing transformant expressed a 4.0±1.0-fold higher level of endogenous EHI_176590 protein (32 kDa, arrows) than did the mock transformant (Fig 3C). Immunofluorescence analysis, at low microscopic magnification (200x), confirmed the uniform expression of EHI_176590-HA in the transformant population, with approximately 71±18% of cells expressing EHI_176590-HA (Fig 3D).

### Overexpression of EHI_176590 increases adhesion to human red blood cells but does not affect motility

Because cell adhesion, phagocytosis, and motility are required for the virulence of *E*. *histolytica*, we next examined whether overexpression of EHI_176590 affects adhesion to human red blood cells (HRBCs); this was conducted using a previously established protocol [7, 29].

After trophozoites of EHI_176590-HA-expressing and mock transformants were incubated with HRBCs on ice for 30 min, the number of HRBCs, bound to a single trophozoite, was counted. Overexpression of EHI_176590 significantly increased the number of adherent HRBCs per ameba, compared with that in the mock transformant; this suggests that EHI_176590 is involved in adhesion (Figs 3E and S4). Similarly, KU27 showed less adhesion to HRBC than did KU50 (S6 Fig). Cell motility on a glass surface was then analyzed by time-lapse imaging. However, we found no significant difference in the average velocity of motility between the EHI_176590-HA-expressing and mock transformants (Fig 3F).

### Association of the EHI_176590 gene with virulence of *E*. *histolytica*

**Fig 4:**
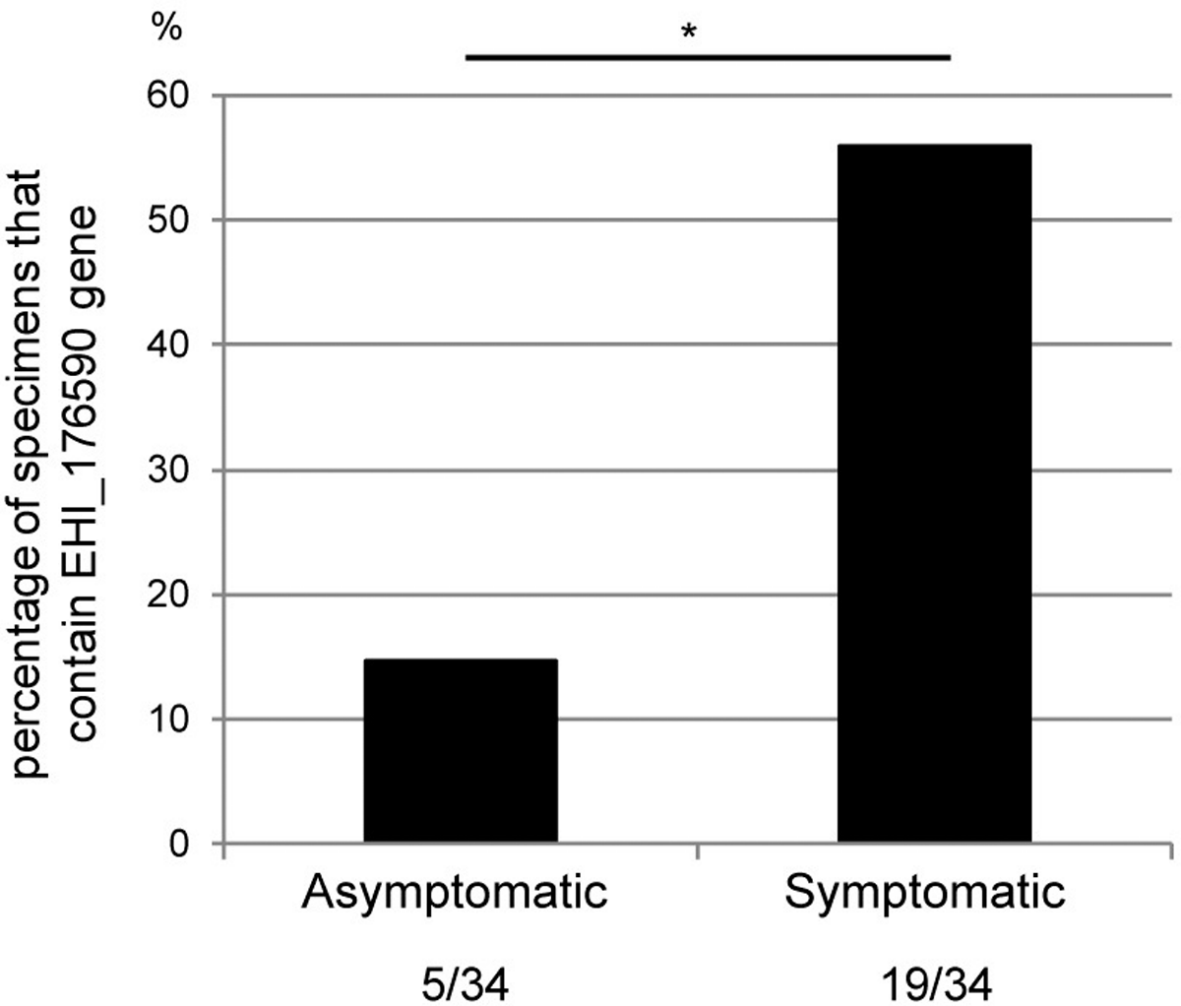
Existence of the EHI_176590 gene in E. histolytica clinical isolates. DNA, extracted from clinical samples (stool or ALA pus) obtained from symptomatic and asymptomatic E. histolytica-infected individuals, was PCR-amplified with EHI_176590 gene-specific primers to examine the presence or absence of the gene. A total of 34 samples, from symptomatic (diarrhea and ALA) and asymptomatic cases, were analyzed. The percentages of PCR-positive samples are shown. *: p-value = 0.0004 by Fisher's exact test.

To determine whether the EHI_176590 gene contributes to the virulence of *E*. *histolytica* in humans, we analyzed the presence or absence of the EHI_176590 gene in *E*. *histolytica* isolated from asymptomatic and symptomatic human clinical samples from Japan and Taiwan. DNA, purified from 34, 16, and 18 stool or abscess samples from asymptomatic, diarrheal, and ALA patients, respectively, was examined for the presence of the EHI176590 gene by PCR (Fig 4). The integrity and quantity of genomic DNA, in the samples, was verified by PCR using control primers designed to amplify NK2 loci of tRNA gene-linked short tandem repeats [17]. The EHI_176590 gene was amplified by PCR and detected in only 14% of the stool samples from asymptomatic humans. In contrast, 56 and 55% of the samples, from diarrheal and ALA cases, respectively, were positive for the gene (p<0.01). These results strongly suggest that the absence of the EHI_176590 gene correlates with the lack of clinical symptoms in infected individuals due to the lack of virulence in the parasite.

### Phylogenetic and structure analysis of *E*. *histolytica* AIG1 family proteins including EHI_176590

Among four differentially present proteins, EHI_176590 and EHI_025990 contain an AIG1 domain (pfam04548), and are, therefore, annotated as AIG1 family proteins. The EHI_176590 gene is followed by two other AIG1 family protein genes, EHI_176580 and EHI_176700; these ORFs are reconfigured by homologous recombination in KU27 (Figs 1C, 2A and 2B). The HM-1 genome encodes 29 AIG1 family proteins including one pseudogene [30]. Maximum likelihood phylogenetic analysis, using 28 AIG1 family proteins, showed that the 28 proteins were grouped into three clusters (Fig 2C), consistent with a previous report [30]. Cluster 1 consists of the most divergent members of this family (Fig 2C). Notably, all AIG1 proteins that displayed differential presence/absence among HM-1, KU27, and KU50 (EHI_176590, EHI_025990, EHI_176580, and EHI_176700) belong to cluster 1 (Fig 2C).

To understand variations in the AIG1 family proteins of other *E*. *histolytica* strains, we obtained AIG1 family proteins from the KU27 genome database in the Amoeba DB and conducted phylogenetic analysis (Fig 2C). KU27 has 27 AIG1 family proteins, which are classified into three clusters. The numbers of genes, belonging to each cluster, in HM-1 and KU27 are: 7 in cluster 1 in both strains; 12 and 13 in clusters 2 and 9, and 7 in cluster 3, respectively. Similar sets of AIG1 family proteins were found conserved in HM-1 and KU27; a unique AIG1 family protein, EMD47880 (EHI5A_208140), generated by homologous recombination in KU27, was classified into cluster 1 (Fig 2C).

Alignment of 28 AIG1 family proteins, in HM-1, revealed unique structural features in the three clusters. Five out of seven cluster 1 proteins had an insertion in the switch II region (S9A Fig). Interestingly, the two exceptions were EHI_176580 and EHI_176700, which flank EHI_176590. To better understand the structural differences between the clusters, we conducted 3D-modeling of representative AIG1 proteins from each cluster: EHI_176590 (Cluster 1), EHI_129470 (Cluster 2), and EHI_022500 (Cluster 3) (S9B Fig). The models were constructed using FORTE [31] with Modeller (9v8), based on alignments between the query and GIMAPs, the AIG1 domain containing protein from mammals (PDB ID codes 2XTP (GIMAP2) and 3ZJC (GIMAP7). Entire folds of the AIG1 domains in these three proteins are conserved, although EHI_176590 has a longer loop region in switch II. We further analyzed the differences between the three clusters and found that at least 54 regions showed biased amino acid cording between clusters (S9C–S9F Fig, S7 Table). Additionally, EHI_176590 has a unique cysteine at position 181 (S9E Fig).

Expression of the 28 AIG1 family proteins, in strains HM-1, KU27, and KU50, were analyzed using a custom-made DNA microarray [24–27] (S5 Fig). Strains HM-1 and KU50 showed a similar pattern of expression; EHI_176590 and EHI_176700 were the two predominately expressed AIG1 family proteins. On the other hand, strain KU27 showed higher expression of EHI_126550. All highly expressed AIG1 family proteins were classified into cluster 1.

### EHI_176590 overexpression increases protrusion formation

**Fig 5:**
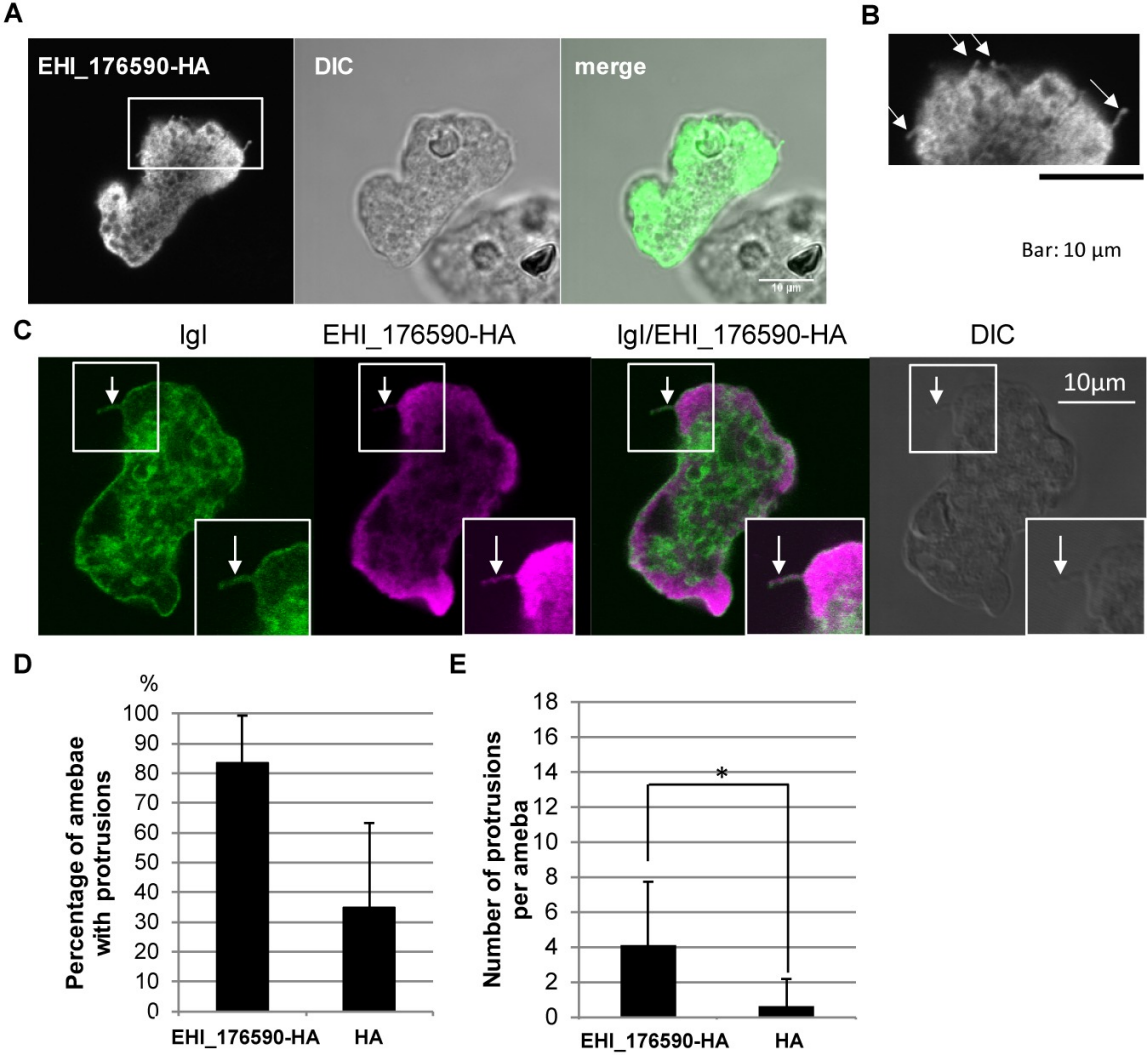
Localization of EHI_175690, and protrusion formation on EHI_175690-HA-expressing amoebae. (A) Immunofluorescence image, obtained by confocal microscopy, showing localization of EHI_176590. EHI_176590-HA-expressing amoebae were fixed and reacted with anti-HA antibody. (B) Magnified image of the inset in (A). Arrows indicate protrusions. (C) Immunofluorescence image, obtained by confocal microscopy, showing localization of EHI_176590-HA and the intermediate subunit of galactose/N-acetylgalactosamine-specific lectin (Igl). EHI_176590-HA-expressing amoebae were fixed and stained with anti-HA and anti-Igl antibodies. Arrow indicates protrusion. Islet showing signal-enhanced image to highlight the protrusion. (D) Effect of EHI_176590-HA expression on the percentage of amoebae that possess protrusions. EHI_176590-HA-expressing and mock transformants (HA) were immunostained as in (C) and the number of cells with protrusions, longer than 0.7 μm, was counted and are presented as percentages. The averages of three independent experiments, in which 100–200 amoebae were counted, are shown. (E) Effect of EHI_176590-HA expression on the number of protrusions per amoeba. EHI_176590-HA-expressing and mock transformants (HA) were immunostained as in (C), and the number of protrusions, per amoeba, was counted. The averages of three independent experiments, in which 100–200 amoebae were counted, are shown. * indicates p<0.01.

To identify the morphological changes, responsible for the enhanced adhesion of trophozoites to HRBCs, and caused by overexpression of EHI_176590-HA, the EHI_176590-HA-expressing transformant was labeled with an anti-HA antibody and examined using confocal microscopy. Immunofluorescence imaging showed that the anti-HA antibody labeled predominantly the cytoplasmic regions, although the labeling was slightly uneven (Fig 5A) compared to the typical cytosolic distribution of ICP1 [32]. We further examined the localization of EHI_176590-HA without membrane permeabilization. The observed labeling pattern of the anti-HA antibody was similar to that observed with permeabilization, but no signal was detected on the plasma membrane (S3 Fig); this suggests that the EHI_176590 protein was not directly involved in adhesion *in situ*.

EHI_176590-HA-expressing amebae also possessed more filopodia, or bleb-like protrusions, than did the control amebae (Fig 5B). To visualize the membrane structures, associated with the protrusions, EHI_176590-HA-expressing amebae were labeled with an antibody against the intermediate subunit of galactose/N-acetylglucosamine-specific lectin (Igl), which is a representative plasma membrane protein (Fig 5C). Immunofluorescence imaging indicated that the protrusions were continuous with the plasma membrane. Both the percentage of amebae, possessing protrusions, and the number of protrusions per ameba, significantly increased by overexpression of EHI_176590 (Fig 5D and 5E). The length of protrusions was comparable between EHI_176590-HA and mock transformants, and the measured lengths were 1.55±0.79 and 1.52±0.64 μm, respectively. These data suggest that EHI_176590 is involved in the formation of plasma membrane protrusions.

To understand the downstream signaling events of EHI_176590, leading to adhesion, we attempted to identify the EHI_176590-interacting proteins by co-immunoprecipitation analysis (S3 Table). Cell lysates from EHI_176590-HA-expressing and mock transformants were used for immunoprecipitation with an anti-HA antibody-conjugated agarose; the resultant immunoprecipitated proteins were analyzed by LC-MS/MS. Aside from ribosomal proteins, second most abundant proteins were EHI_176580 and EHI_176700, the AIG1 family proteins, which are encoded by the genes upstream and downstream of the EHI_176590 gene (Fig 1C). We also identified a TBC domain containing RabGAP, two Igl2 proteins, calcium binding family protein (CaBP), and a diaphanous protein. CaBP and diaphanous protein may be involved in the rearrangement of the actin cytoskeleton (see Discussion). However, because of the low level of detection, these proteins were not further characterized.

**Fig 6:**
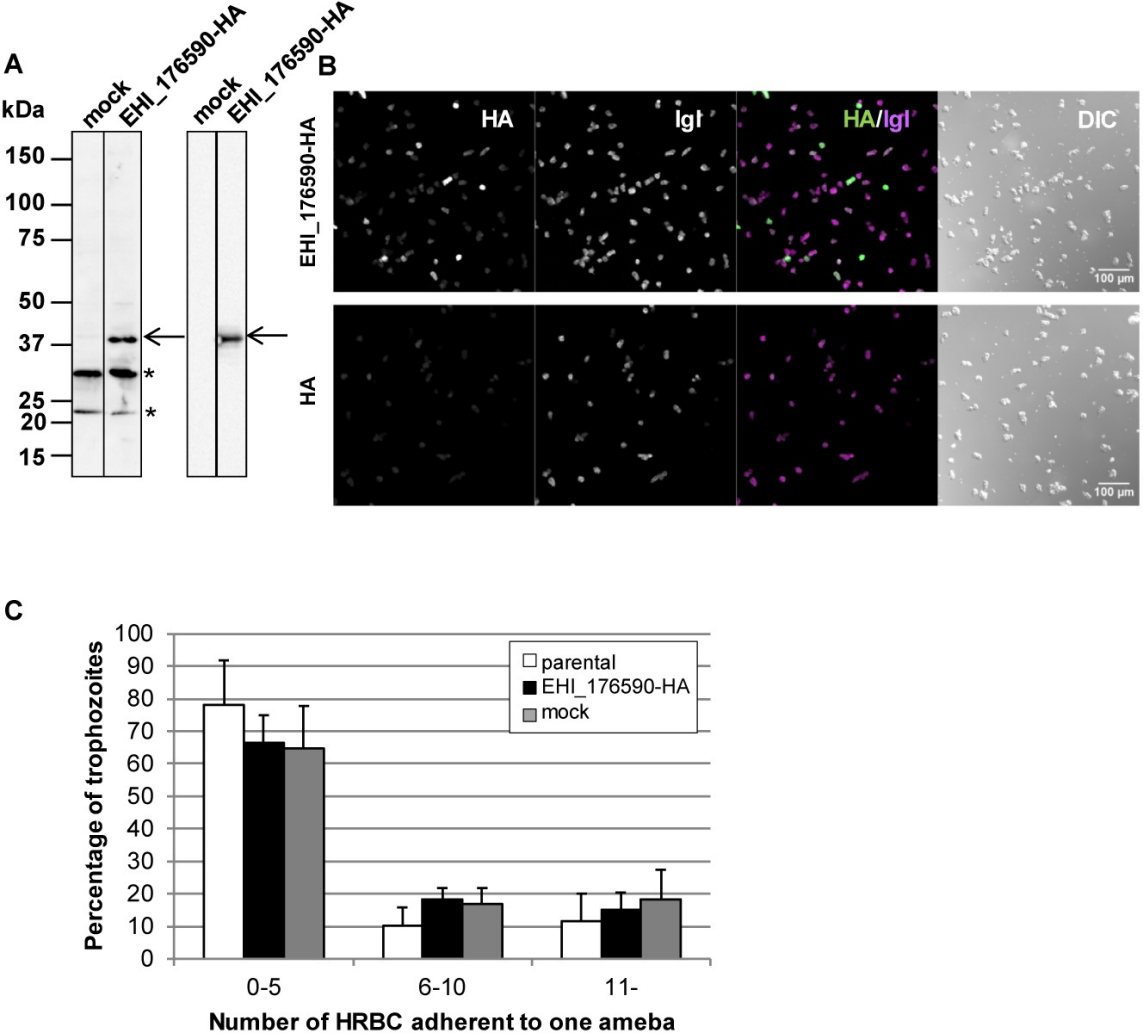
**EHI_176590-HA expression in KU27** (A) Immuno-detection of EHI_176590-HA ectopically expressed in strain KU27. Total cell lysates, from mock-transfected and EHI_176590-HA-expressing strain KU27 trophozoites, were analyzed by immunoblot analysis with anti-EHI_176590 (left panel) and anti-HA antibody (right panel). The arrow indicates EHI_176590-HA, and asterisks indicate protein bands detected by non-specific immunoreactivity. (B) Low-magnification confocal immunofluorescence images of EHI_176590-HA-expressing and mock-transfected KU27. Trophozoites were fixed and reacted with anti-Igl and anti-HA antibodies. Merged fluorescent (IGL/HA) and DIC images are also shown. (C) Adhesion of the parental, EHI_176590-HA-expressing, and mock-transfected KU27 to HRBCs. Parental (open bars), EHI_176590-HA-expressing (black bars), and mock-transfected (gray bars) KU27 strains were co-cultured with HRBCs on ice for 30 min, and adherent HRBCs per ameba were counted. The total number of trophozoites was set to 100%, and the percentage of trophozoites, bound to 0–5, 6–10, or >10 HRBCs, are shown. Error bars indicate standard deviations of four biological replicates.

Next, we examined the correlation between the lack of EHI_176590 expression and reduced adherence of strain KU27 trophozoites to HRBCs. For this, we examined whether the expression of EHI_176590, in KU27, restored adhesion to HRBCs. We generated an EHI_176590-HA-expressing strain of KU27 (Fig 6A) and determined that 36.2±2.4% of cells expressed EHI_176590-HA (Fig 6B). The ability of EHI_176590-HA-expressing KU27 to adhere to HRBCs was comparable to that of parental KU27 and the mock transformant. Approximately 80% of the amebae, from the parental, mock, and EHI_176590-HA-expressing KU27 lines, adhered to 0–5 HRBCs (Fig 6C). Taken together, these data indicate that EHI_176590 is not the sole determinant/regulator of adhesion in KU27.

### Gene silencing and overexpression of EHI_176590 conversely affect *E*. *histolytica* virulence in a liver abscess model

**Fig 7:**
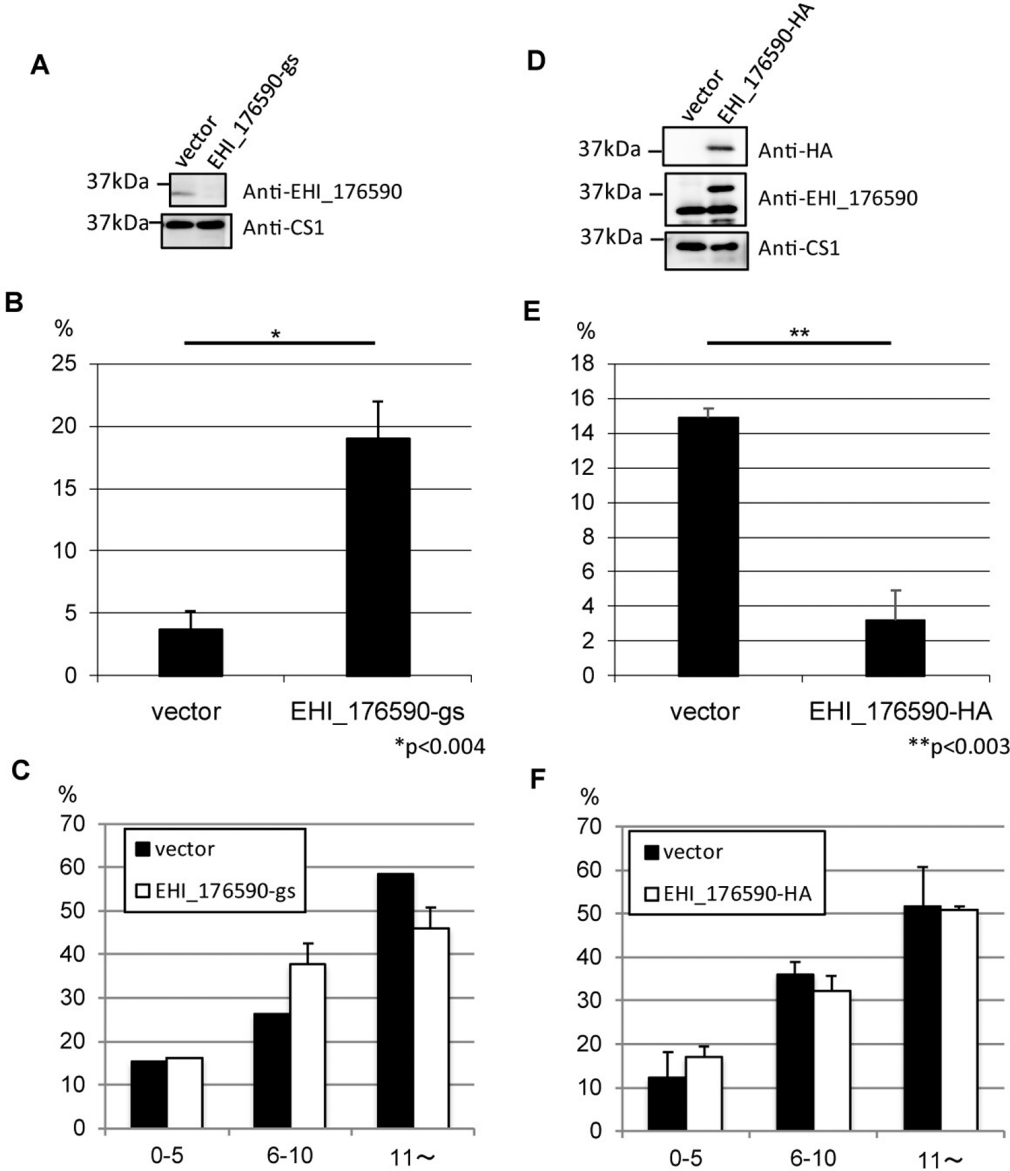
Liver abscess formation in hamsters challenged with EHI_176590 gene-silenced and EHI_176590-HA-expressing transformants using animal-passaged virulent strain Cl6. (A) Confirmation of EHI_176590 gene silencing by immunoblot analysis. Total lysates, from EHI_176590 gene-silenced and mock transformants, were subjected to immunoblot analysis using anti-EHI_176590 (upper panel) and control anti-CS1 antibodies (lower panel). (B) Effect of EHI_176590 gene silencing on liver abscess formation. Approximately 1×10^6^ trophozoites of EHI_176590 gene-silenced and mock-transfected strains (created from animal-passaged virulent Cl6) were injected into the livers of hamsters. Injected animals were sacrificed 6 days post-infection, and the liver and abscesses were dissected and weighed separately. Averaged percentages of the weight of abscesses per liver are shown. *p<0.004. (C) Adhesion of EHI_176590 gene-silenced and mock-transfected strains to HRBCs. Mock-transfected (black bars) and EHI_176590 gene-silenced (white bars) strains were co-cultured with HRBCs on ice for 30 min, and adherent HRBCs per ameba were counted. The percentage of trophozoites, bound to 0–5, 6–10, or >10 HRBCs, are shown. Error bars indicate standard deviations of three biological replicates. (D) Confirmation of EHI_176590-HA expression by immunoblot analysis. Total lysates from EHI_176590-HA and mock transformants were analyzed by immunoblot with anti-HA, anti-EHI_176590, and anti-CS1 antibodies (upper, middle, and lower panels, respectively). (E) Effect of EHI_176590-HA overexpression on liver abscess formation. Approximately 3×10^6^ trophozoites, of EHI_176590-HA and mock-transfected transformants, were injected into the liver of hamsters, and liver abscess formation was evaluated as in (B). **p<0.003. (F) Adhesion of EHI_176590-HA and mock-transfected strains to HRBCs. Trophozoites of mock-transfected (black bars) and EHI_176590-HA transfected (white bars) strains were co-cultured with HRBCs, and adhesion was evaluated as in (C).

Next, we investigated the apparent causal relationship between the level of EHI_176590 expression and *in-vivo* virulence of *E*. *histolytica*. For this, we generated a hamster liver abscess model by infecting animals with the ameba transformant lines in which the EHI_176590 gene was either transcriptionally silenced or overexpressed. For these animal experiments, we used the hamster liver-passaged virulent Cl6 strain and a gene silencing system originally developed by the Singh group [33]. Gene silencing of EHI_176590, and expression of EHI_176590-HA, were confirmed by immunoblotting (Fig 7A and 7D). EHI_176590 gene-silenced and EHI_176590-HA-overexpressing trophozoites were directly injected into the liver of hamsters (1×10^6^ and 3x10^6^ cells/animal, respectively). The average weight of abscesses, produced by the EHI_176590 gene-silenced strain, was 5-fold higher than that produced by the control strain (Fig 7B). In contrast, the average weight of abscesses, produced by the EHI_176590-HA-overexpressing strain, was 4.6-fold lower than that produced by the control strain (Fig 7E). The adhesion of these strains to HRBCs was comparable between the EHI_176590 gene-silenced and control strains, and between the EHI_176590-HA-overexpressing and control strains (Fig 7C and 7F).

## Discussion

### Identification of a potential virulence-associated gene by comparative genomic analysis

To our knowledge, this is the first report describing the loss of virulence in *E*. *histolytica* following a genomic recombination event; this was associated with the loss of the EHI_176590 gene, and was identified by comparative-genomic analysis of two representative Japanese clinical isolates. We detected a possible causal connection between the presence of the EHI_176590 gene and virulence because the encoded protein appeared to be involved in the formation of filopodia, or bleb-like protrusions, on the plasma membrane. Furthermore, we demonstrated that this gene is commonly deleted in clinical strains isolated from asymptomatic cases, but not in strains from symptomatic cases. The deletion of this genomic region can occur by a single homologous recombination event (Fig 2). The presence and mechanism of homologous recombination in *E*. *histolytica* is not well demonstrated. However, previous studies strongly suggest the existence of this process [20, 34]. Indeed, the genome of *E*. *histolytica* contains numerous repetitive elements, including SINEs or LINEs, but prediction of recombination hot spots is presently not possible. We examined whether the regions of this recombination event are present in other locations in the genome; however, we failed to detect any such regions having significant similarity (over 90% identity to the 194-bp homologous region flanking EHI_176590 ORF) to EHI_176590. Furthermore, the contig, containing EHI_176590, contains none of SINE, LINE, or other known repetitive sequences implicated in homologous recombination.

### AIG1 family proteins identified by previous comparative genomic analysis

In a previous study, Weedall and colleagues conducted comparative genomic analysis of five colitis and three asymptomatic strains of *E*. *histolytica* [20], and we found that the two genes (EHI_089440 and EHI_176590) missing in strain KU27 are also absent in two (IULA:1092:1 and HK-9) and three (HK-9, PVBM08B, and PVBM08F) strains, respectively, of these eight strains, including those isolated from colitis cases. In addition, these two genes are present in two Bangladeshi strains from asymptomatic cases and in a Rahman strain [20]. In our study, several clinical samples, obtained from asymptomatic cases, also lacked the EHI_176590 gene, although the detection rate of the gene was significantly higher in symptomatic cases than that in asymptomatic cases (Fig 4). There are three possible explanations for this finding. First, the *E*. *histolytica* strains, isolated from asymptomatic patients, may not be low-virulence strains; rather, it is possible that the patient was not exhibiting discernible clinical symptoms at the time of stool sample collection [35]. Second, the strains were isolated from asymptomatic cases in Bangladesh and the U.K. [20], and Japan and Taiwan; therefore, sampling may have been subject to geographical bias. Third, it is possible that the EHI_176590 gene was not detected in 44% of clinical samples, isolated from symptomatic cases, because the symptoms may have been caused by infections other than *E*. *histolytica*.

### EHI_176590 was previously identified as a virulence-associated gene by comparative transcriptomics

Numerous comparative-transcriptomics studies, aimed at finding virulence-associated genes in *Entamoeba*, were conducted using known virulent and non-virulent species or strains, or after animal-challenge experiments or exposure to human tissue explants [10–12, 22, 23, 36]. Using this approach, several genes, encoding various AIG1 family proteins, were found to be differentially expressed and, thus, were implicated in virulence. Several genes, encoding AIG1 family proteins, have been removed from the current *E*. *histolytica* genome database because of artefactual tandem duplications of genes in the initial database [30]; thus, several genes, identified in previous reports, were removed from Amoeba DB, but remain in the NCBI.

Transcriptomic comparisons, between virulent HM-1 and low-virulence Rahman strains, showed that XP_653207 and XP_648101 (NCBI accession numbers are provided when the genes are only available in the NCBI and have been deleted from Amoeba DB) were more highly expressed in HM-1; however, EHI_176590 had similar levels of expression [23]. A comparison of two isogenic HM-1 lines was used to examine a low-virulence line A and a virulent line B, which differ in virulence in gerbils. This analysis indicated that 18 AIG1 family protein genes, including EHI_176590 and EHI_176580, were more highly expressed in the virulent line B, whereas only one AIG1 family protein gene, XP_648127, was more highly expressed in the low-virulence line A [12]. In the murine colon, the expression levels of 12 AIG1 family protein genes, in HM-1, significantly change between days 1 and 29 post-infection [10]. Among these 12 genes, 10 genes show higher expression at day 1 compared with that at day 29. For example, the expression of EHI_176590 is upregulated 2.1-fold on day 1 and downregulated 2.5-fold on day 29, compared with that at day 0. In colon explants, the expression of EHI_176700 was also upregulated when HM-1 trophozoites were exposed to colon tissue for 1 h [36]. The fact that EHI_176590 expression was upregulated in a highly virulent strain [12], and during the initial stage of cecal infection [10], supports our finding that EHI_176590 is involved in virulence. In a recent study, Weber and colleagues conducted a comprehensive RNA-seq analysis of virulent HM-1, exposed to different conditions, such as *in-vitro* culture (virulence potentially attenuated), coculture with human colon explants, and *in-vivo* challenge using the hamster liver abscess model [13]. Interestingly, the expression of EHI_176580, EHI_176590, and EHI_176700 was reduced in the amebae recovered from liver abscesses, but remained unchanged in the amebae coincubated with human colon. There are several other AIG1 family genes whose expression was dependent on environmental changes (S6 Table); this suggests that the expression of EHI_176590, and proximal AIG1 family protein genes, is a response to invasion of the liver. Taken together, these results indicate that AIG1 family proteins, in *E*. *histolytica*, are involved in pathogenesis and stress responses during infection via interactions with mammalian tissues (see below). It is also possible that each *AIG1* gene is involved in a distinct biological process.

Recent evidence suggests that AIG1 family proteins are involved in stress responses. For example, the treatment of *E*. *histolytica* with the nitric oxide releaser, DPTA, causes a 7.4-fold reduction in the expression of EHI_089670 [37]. In addition, treating a metronidazole-resistant strain with metronidazole causes up- and down-regulation of five and four AIG1 family proteins, respectively [24]. Furthermore, the genes encoding three AIG1 family proteins, including EHI_089670, are exclusively expressed during the cyst stage, which is considered to be a stress-responsive developmental stage [38]. Taken together, these results suggest that AIG1 family proteins, in *E*. *histolytica*, are involved in environmental stress responses. Heterogeneity, among the various AIG1 family protein genes, present in strains of *E*. *histolytica*, may cause inter-strain differences in response to environmental and host factors, and may be associated with virulence in the host.

### Heterogeneity and roles of AIG1 family proteins in *E*. *histolytica*

The gene, encoding AIG1, which is a GTPase domain-containing protein, was originally identified as being upregulated in a strain of *Pseudomonas syringae* pv *maculicola* following infection of *Arabidopsis thaliana;* this gene may, therefore, be involved in immune responses in plants [39]. AIG1 family proteins may also be involved in the development of *A*. *thaliana* and its responses to environmental stimuli [40]. Orthologous genes of AIG1 family proteins include GTPases of immunity-associated protein (GIMAPs)/immune-associated nucleotide-binding protein (IAN) family proteins, which are conserved in mammals and are involved in T- and B-cell development via interactions with Bcl-2 family proteins [41–44]. Recently, the function of GIMAPs was attributed to their oligomerization ability [45, 46]. Structural analyses also revealed that GIMAPs are classified in the TRAFAC (translation factor associated) class of GTPases; members of this class form oligomers similar to dynamin, translocase of chloroplast (Toc), and septin [47]. The association of GTP with GTPase-inactive GIMAP may induce oligomerization of GIMAP, forming scaffolds for interacting partners such as Bcl-2 family proteins; these oligomers become destabilized by association with GTPase-active GIMAP [48]. Several GIMAPs are also associated with the cytoskeleton and are involved in calcium influx after T-cell receptor activation and IFN-γ secretion from CD4^+^ T cells [49, 50].

Biller and colleagues identified 47 AIG1 family proteins with an AIG1-like GTPase domain in *E*. *histolytica* [12], and the latest reannotation of the genome confirmed the presence of 29 AIG1 family proteins [30]. The AIG1 family of proteins is one of the largest protein families; the members of this family are physically linked to transposable elements [30]. A unique feature of AIG1 family proteins in *E*. *histolytica* is that only the first three (C1, C2, and C3) of five GTP-binding sites, in the putative GTPase domain, are conserved. *In-silico* analyses suggest that the genes, encoding AIG1 family proteins, may be uniquely regulated and have novel functions in *E*. *histolytica*.

The results of our phylogenetic analysis show that AIG1 family proteins in *E*. *histolytica* are grouped into three major clusters; EHI_176590, and the two adjacent AIG1 family proteins (EHI_176580 and EHI_176700), are grouped in the same cluster (cluster 1; Fig 2C). Numerous genes, and a variety of AIG1 family proteins, are nearly conserved in strain KU27 (Fig 2C); however, there are 8 and 7 HM-1- and KU27-specific AIG1 family proteins, respectively. This analysis suggests that AIG1 family proteins play unique and important roles in each strain of *E*. *histolytica*. In the *E*. *histolytica* HM-1 genome, the genes, encoding AIG1 family proteins, are present in a tandem array of 2–3 genes in four regions. Notably, AIG1 family protein genes, which comprise each of the four clusters of tandemly arrayed genes, belong to a different cluster (S4 Table). These phylogenetic and genomic structural data suggest that the AIG1 family protein gene tandem array, containing the EHI_176590 gene, was likely recently established by the events of gene duplication.

Multiple sequence alignment analysis and 3D-modeling revealed significant differences between the three clusters of AIG1 family proteins in *E*. *histolytica* (S9 Fig and S7 Table). Cluster1 proteins, including EHI_176590, have a longer switch II region. The switch II region, of the AIG1 domain, associates with helix 7 in the GDP-binding or nucleotide-free status, and is also used to avoid oligomerization [51]. However, the conservation of helix 7 in amoebic AIG1 family proteins is promiscuous. Only EHI_176590 has a cysteine at 181, which may be involved in unique disulfide bonds with proximal cysteine at 138. Further studies will determine the structure-function relationships of AIG1 family proteins in *E*. *histolytica*.

### How is the AIG1 family protein involved in the virulence mechanism of *E*. *histolytica*?

We have shown that EHI_176590 is involved in the formation of surface protrusions in *E*. *histolytica*. The observed protrusions had a similar size and structure to those of *E*. *histolytica* filopodia [52–55], which are 1–10 μm in length and 0.3 μm in diameter [53]. Amebic filopodia are often observed along the side of the adherent surface and presumably strengthen adhesion to prey [52]. In general, filopodia are rich in actin filaments, which consist of F actin [56]; we did not, however, detect F actin by phallacidin staining in the protrusions (S10 Fig). Further study is needed to characterize this structure.

Filopodium-associated adhesion of *E*. *histolytica* trophozoites to MDCK [52], Caco-2 cells [54], and the mucin layer of human colon explants [55] has been observed by scanning electron microscopy. Consistent with those observations, overexpression of EHI_176590-HA, in strain HM-1, enhanced adhesion to HRBCs, confirming a causal link between formation of protrusions/filopodia and adherence. The importance of filopodia in adhesion was previously reported in *Dictyostelium discoideum* and *Acanthamoeba castellanii* (acanthopodia) [57], in which filopodium dynamics are regulated by the diaphanous-related formin dDia2 [58–60]. Genes, encoding diaphanous domain-containing formins, are conserved in the genome of *E*. *histolytica*. Immunoprecipitation of EHI_176590 identified a diaphanous protein homolog that was reproducibly associated with EHI_176590 (S3 Table). Additionally, one CaBP, CaBP20 [61], previously annotated as a myosin light chain, was also detected as an EHI_176590-associated protein (S3 Table). Also, myosin X in mammalian cells, is known to be involved in filopodia formation [62, 63]. These data support the notion that EHI_176590 is involved in filopodia formation via interaction with diaphanous proteins, thereby positively regulating adhesion to host cells and tissues. However, because overexpression of EHI_176590-HA did not enhance adhesion to HRBCs in KU27, other factors may also regulate HRBC adhesion and protrusion formation in *E*. *histolytica*. In addition, other, as of yet unidentified, genomic differences may be responsible for the observed variances in adhesion between strains KU27 and KU50. Numerous unique single nucleotide polymorphisms exist between the two strains, which supporting this speculation.

### Overexpression and gene silencing of EHI_176590 exert opposite effects on liver abscess formation and virulence hallmarks *in vitro*

Gene silencing and overexpression of EHI_176590 enhanced and reduced the size of liver abscesses, in the experimental animals, respectively; this observation was counter-intuitive. First, a reciprocal correlation was observed between the expression level of EHI_176590 and abscess size. Second, the effect of EHI_176590 overexpression on adhesion to HRBCs varied depending on cell line. Third, no correlation was observed between the ability to adhere to HRBCs and abscess size. Using a gerbil liver abscess model, Meyer and colleagues also showed that among the 12 clones of an attenuated low-virulence HM-1:IMSS A strain and animal-passaged virulent HM-1:IMSS B strain, there was no strong correlation between *in-vitro* virulence phenotypes and *in-vivo* virulence in gerbils. This suggests that independent mechanisms are involved in regulation of pathogenicity in *E*. *histolytica* [64]. Comprehensive phenotypic and transcriptomic analyses of a representative non-pathogenic A clone (A1^np^), pathogenic B clone (B2^p^), and non-pathogenic B clone (B8^np^) indicated that *in-vitro* characteristics, such as activity of cysteine peptidase, hemolytic activity, erythrophagocytosis, motility, and cytopathic activity, previously considered to be indicative of virulence, do not correlate with liver abscess size in gerbils. In addition, gene expression profiles of A1^np^ and B8^np^ markedly differ, despite the isogenic nature of the clones; this highlights the difficulty of using transcriptomic data to identify common genes and mechanisms of repression (or attenuation) involved in virulence. These observations are consistent with the notion that virulence is not regulated by a single gene, but rather a gene circuit, in *E*. *histolytica*.

Here, we used several strains, which potentially differ with respect to their genetic/epigenetic background (i.e., wild-type Cl6, hamster liver-passaged Cl6, G3, and clinical strains) to evaluate virulence. We found that the contribution of EHI_176590 to protrusion formation, adhesion to HRBCs, and formation of liver abscesses varied depending on the presence or absence of other factors. We have also shown that in mammalian cells, the AIG1 gene had various effects depending on the status of the cells or experimental conditions. For example, transient overexpression of GIMAP5, in cultured naïve T cells, enhanced apoptotic cell death; however, no similar effect was observed in activated T cells [65]. This may be caused by the low expression level of GIMAP5 in naïve T cells and high expression level in activated T cells [65, 66].

### Is EHI_176590 a major determinant of virulence or a surrogate marker for virulence in *E*. *histolytica*?

EHI_176590 gene deletion was confirmed in 86 and 40% of the samples, obtained from asymptomatic and symptomatic cases, respectively. We cannot exclude the possibility of a discrepancy between declared symptoms and histology. It is necessary to unequivocally demonstrate a causal relationship between EHI_176590 and virulence in a future study. Strains, lacking EHI_176590, need to be confirmed for loss of virulence in humans or human-derived tissue/organ models, such as *ex-vivo* human colon model or colon organoids. Our results indicate that the EHI_176590 gene is not present in high proportion in low-virulence clinical isolates from Japan and Taiwan. To our knowledge, this is the first report to show that a gene, identified by comparative genomic analysis, is involved in virulence-related biological processes such as protrusion formation and adhesion. Further studies are needed to understand the underlying molecular mechanisms by which EHI_176590 contributes to these processes.

## Materials and methods

### *E*. *histolytica* strains, cultivation, and clinical samples

Two previously isolated and characterized Japanese *E*. *histolytica* isolates (KU27 and KU50, obtained from Kieo University) were used for comparative genomics [15, 16, 21]. KU27 was collected from an asymptomatic individual in 2001, and KU50 was isolated from a diarrheal/dysenteric patient [21]. Trophozoites of KU50 were maintained axenically at 35.5°C, in 13 × 610 mm screw-capped Pyrex glass tubes or 25 cm^2^ tissue culture flasks (#152094; Nunc, Roskilde, Denmark), in yeast extract-iron–maltose-dihydroxy-acetone-serum (YIMDHA-S) medium [67, 68]. Trophozoites of KU27 were cultured monoxenically with *Crithidia fasciculate*, in YIMDHA-S medium, using the same tubes/flasks as those used for strain KU50. Trophozoites of *E*. *histolytica* strain HM-1:IMSS cl6 (Cl6) [69] (wild type, maintained *in vitro* >10 years and attenuated) and G3 [70] were cultured axenically in BI-S-33 medium [71]. Concurrently, virulent trophozoites of Cl6, which were recovered from liver abscesses of golden hamsters [72], were cultured monoxenically in YIMDHA-S medium co-cultured with *Crithidia fasciculata* [67, 68]. Ameba transformants were cultured in the presence of geneticin (Life Technologies, MD, USA). All trophozoites were harvested during the exponential growth phase (approximately 48 h) and collected by centrifugation at 300 × g for 5 min. Clinical samples, collected at Keio University and Tokai University, were anonymized, and only extracted DNA samples were provided. Clinical samples, collected by Taiwan CDC, were used at Taiwan CDC, and only PCR results for EHI_176590 ORF and NK2 locs were provided. We examined stool samples from 34 and 16 asymptomatic and diarrheal cases, respectively, in Japan and Taiwan. Among the 34 asymptomatic cases, 5 samples were from Japan and 29 were from Taiwan. Among the 16 diarrheal cases, 11 samples were from Japan and 5 were from Taiwan. We also examined 18 liver abscess aspirates, which were collected from 16 ALA patients in Japan and 2 ALA patients in Taiwan.

### Ethics statement

Two *E*. *histolytica* strains, KU27 and KU50, were provided by the Keio University School of Medicine, Japan, as established axenic cultures. Stool samples were collected at Keio University. Samples of liver abscess aspirates were collected at Tokai University School of Medicine, Japan. Stool and liver aspirate samples were also collected at Taiwan CDC, Taiwan. Human red blood cells were obtained from healthy volunteers at the National Institute of Infectious Diseases (NIID), Japan. All clinical samples were anonymized. The use of clinical samples from Taiwan, in this study, was approved by the ethics committee of the Taiwan CDC (IRB 103104). All individuals provided written informed consent for the use of collected samples.

Protocols for the animal experiments, and the use of hamsters, were approved by the Animal Use Committee of NIID under the guide for animal experiments performed at NIID (registration number, 116042). All animal care procedures were in accordance with Standards Relating to the Care and Management of Laboratory Animals and Relief of Pain formulated by Ministry of the Environment.

### Genomic DNA isolation and sequencing

Genomic DNA, from strains KU27 and KU50, was extracted using a QIAamp DNA Mini Kit (Qiagen, Hidden, Germany), according to the manufacturer’s instructions. Trophozoites of KU27 and KU50 were cultured in 25 cm^2^ tissue culture flasks (2–6 flasks) to obtain 4 to 9 μg of genomic DNA. Approximately 4–5×10^6^ amebae were obtained per flask. Purified genomic DNA samples were subjected to next-generation sequencing using an Illumina Genome Analyzer IIx DNA Sequencer (GAIIx; Illumina, San Diego, CA). Genomic DNA libraries were constructed using a genomic DNA Sample Prep Kit (Illumina). DNA clusters were generated on a slide, using a Cluster Generation Kit v4 on an Illumina cluster station, according to the manufacturer’s instructions. All sequencing runs, for 125-mers, were performed using an Illumina GAIIx and Illumina Sequencing Kit (v5).

### Read mapping and comparative genome analysis

To compare genome sequences of KU27 and KU50, short, paired-end reads of KU27 and KU50 were mapped onto a reference genome of *E*. *histolytica* HM-1: IMSS using bwasw of bwa (v 0.6.1) [73] and samtools (v 0.1.18) [74] at default parameters. Mapped read counts of each gene were calculated by bedtools (v. 2.17.0) [75] and normalized by reads per kilobase of exon per million mapped reads (RPKM) according to the method by Weedall et al [20]. The mapping data were visualized with GenomeJack viewer software (v 3.1) (Mitsubishi Space Software, Tokyo, Japan) (http://genomejack.net/english/index.html). The predicted gene copy number was calculated at RPKM score of each gene divided by median of all RPKM. To investigate the phylogenetic relationship between KU27 and KU50, SNV phylogenetic analysis was performed on the genomes of *E*. *histolytica*, which were deposited into the GenBank database as follows. To construct simulated paired-end reads from the available genomic sequences of seven *E*. *histolytica* strains, SimSeq software [76] was used with the following default parameter modifications: 14,000,000 paired-end reads, mean insert size of 200 mer, read length of 150 mer, and no mutations model. The short read mapping data were constructed as described above. All variant sites were extracted by VarScan v2.3.4 [77] using default parameters; the biallele sites, which were associated with polyploidy, were removed. The variant sites, on the repeat regions of the HM-1 genome, which were detected by NUCmer [78], were also excluded from single nucleotide variations (SNVs) phylogenetic analysis because those variant sites are considered unreliable. All SNVs, on the core genome, were concatenated to generate a pseudo sequence. The DNA maximum-likelihood program (RAxML v7.25) [79] was used for phylogenetic analysis with 1,000-fold bootstrapping. FigTree v. 1.2.3 software was used to display the generated tree.

### Amplification and sequencing of deleted genomic regions

Genomic regions, corresponding to NW_001915296, were amplified by PCR using PrimeSTAR GXL DNA polymerase and a Veriti thermal cycler (Life Technologies, MD, USA). Two primers, #107 (5’- CAACACACAACTATCAGATTCTTTTGATTC-3’) and #121 (5’- CCAATATAATACCTTACTTATTCATGATAC-3’) were used for the amplification reaction. Approximately 10 ng of genomic DNA, from KU27 and HM-1, was used as template.

### Multiple alignments, phylogenetic analysis, and 3D-modeling

Multiple alignment for nucleotide sequences was performed using BLASTN homology search, at default parameters, followed by visualization of the aligned images with ACT [80]. For phylogenetic analysis of AIG1 family proteins in strains HM-1 and KU27, multiple alignment for amino acid sequences was performed by mafft v6.86 [81] using L-INS-i parameters, followed by phylogenetic analysis using Maximum likelihood method in RAxML v7.25 [79] with 1,000 bootstrap iterations. FigTree v. 1.2.3 software was used to display the generated tree.

To elucidate structural differences between the three clusters of AIG1 family proteins in strain HM-1, entire sequences of the 28 AIG1 family proteins were aligned using MAFFT (v7.351b) [82], and visualized using MSAViewer [83] or NCBI Multiple Sequence Alignment Viewer 1.6.0.

3D-models of AIG1 domains in EHI_176590, EHI_129470, and EHI_022500 were constructed using FORTE [31], with Modeller (9v8) based on alignments between the query and GIMAPs, AIG1 domain containing protein from mammals (PDB ID codes 2XTP (GIMAP2), and 3ZJC (GIMAP7).

### Plasmid construction and *E*. *histolytica* transformants

To express *EHI_176590* with a carboxyl-terminal hemagglutinin (HA) tag *(EHI_176590*-HA) in ameba trophozoites, the protein-coding region of EHI_176590, lacking the stop codon, was amplified by PCR from cDNA using primers 5’-ACCAGATCTATGAGTATCGAAGAAGTAAAA-3’ and 5’-GTTAGATCTATACATCAGCCTATCCTGAGT-3’ (BglII restriction sites are underlined); it was then subcloned into BglII-digested pEhExHA [84] (*pEHI_176590*-HA).

To generate a gene-silenced ameba line in strain G3, a 420-bp long fragment, corresponding to the amino terminus of the *EHI_176590* protein-coding region, was amplified by PCR from cDNA using primers 5’-CATAGGCCTATGAGTATCGAAGAAGTAAAA-3’ and 5’-GCGGAGCTCATTATAACACATAGTCCATAC-3’ (StuI and SacI restriction sites are underlined); it was then subcloned into StuI/SacI-digested pSAP2-Gunma [85] (pSAP2-EHI_176590).

To generate an EHI_ 048600 gene-silenced strain in liver-passaged Cl6 background and retain the ability to produce amoebic liver abscesses in hamsters, we first cloned a 130-bp region, corresponding to the amino-terminal portion of EHI_ 048600 protein-coding sequence [33], into pEhEx [86]. The fragment was amplified by PCR from cDNA using primers 5-GACAAACACATTAACATGGAAATTGAATTAACCCTC-3’ and 5’-AAAAGAAGAGTTCAACTCGAGCCCGGGAGATCTCGTTGATGCTGCAATTTTTG-3’, and was then subcloned into BglII/XhoI-digested pEhEx using In-Fusion HD Cloning kit (Takara, Shiga, Japan). The resulting plasmid, designated pEhEx-04trig, contained multiple restriction sites (BglII, SmaI, and XhoI) downstream of the 130-bp region of EHI_ 048600. The entire protein-coding region of the *EHI_176590* gene was amplified by PCR, from pEhEx-EHI_176590-HA, using primers 5’-GGGAGATCTATGAGTATCGAAGAAGTAAAA-3’ and 5’-ATGCTCGAGCTAATACATCAGCCTATCCTG-3’; it was then subcloned into the BglII/XhoI-digested pEhEx-04trig to generate pEhEx-04trig-EHI_176590.

pEHI_176590-HA was introduced into *in vitro*-maintained (thus, potentially attenuated) Cl6 and hamster-liver passaged virulent Cl6, by liposome-mediated transfection as previously described [87]; this created the overexpressing transformant lines. pSAP2-EHI_176590 and pEhEx-04trig-EHI_176590 were similarly introduced into strain G3, and the virulent Cl6 line, respectively, to create transformant lines with repressed expression of the EHI_176590 gene. Geneticin was added, at a concentration of 3 μg/mL, 24 h after transfection; the concentration of G418 was gradually increased for approximately 2 weeks until reaching 10 μg/mL for Cl6 or 6 μg/mL for the G3 strain. To establish EHI_176590-HA, expressing KU27, *pEHI_176590*-HA was introduced into strain KU27 using lipofectamine. For this transfection, the co-cultivation period was reduced to 1 h, and geneticin was added at a concentration of 1 μg/mL 24 h after transfection.

### *E*. *histolytica* strains used

To generate overexpressing and gene silenced transformants, the following combinations of *E*. *histolytica* strains and plasmids were used:

To express EHI_176590-HA for analysis of *in-vitro* phenotypes: strain, cl-6, pEhExHA-EHI_176590, and pEhExHA (control).

To express EHI_176590-HA to examine virulence in hamsters: liver-passaged cl-6, pEhExHA-EHI_176590, and pEhExHA (control).

To express EHI_176590-HA to examine the phenotype in KU27: KU27, pEhExHA-EHI_176590, and pEhExHA (control).

To gene silence EHI_176590 to examine antibody specificity: G3, pSAP2-EHI_176590, and pSAP2-gunma (control).

To gene silence EHI_176590 to examine virulence in hamsters: liver-passaged cl-6, pEhEx-04trig-EHI_176590, pEhEx-04trig (control).

### Antibodies

Peptide antibodies against EHI_176590 were raised against a mixture of KLH-conjugated synthetic peptides DVTDDVNSKTQKTSGFY (corresponding to a.a. 35–51 of EHI_176590) and KPIPDQYSNTSKEGFEK (a.a. 279–295), in rabbits, at MBL (Nagoya, Japan). The obtained anti-sera were further affinity-purified on a column containing a resin coupled with a peptide immunogen; this procedure was conducted at MBL. Anti-HA.11 (clone 16B12) mouse monoclonal antibody was purchased from COVANCE (Berkeley, CA, USA). Anti-HA rabbit polyclonal antibody was purchased from MBL. Alexa Fluor-conjugated anti-mouse and anti-rabbit IgG goat antibodies were purchased from Life Technologies. Horseradish peroxidase-conjugated anti-mouse goat and anti-rabbit donkey antibodies were purchased from Thermo Scientific (Hudson, NH, USA). The mouse monoclonal antibody against Igl was a kind gift from Dr. Hiroshi Tachibana (Tokay University, Kanagawa, Japan)[88].

### Immunoblot analysis

Whole cell lysates were analyzed by SDS-polyacrylamide electrophoresis (PAGE) and immunoblotting, as previously described [84]. The dilution of the primary antibodies was 1:200 for anti-EHI_176590 antibody and 1:1000 for anti-HA antibody.

### Immunofluorescence assay and filopodia counting

Immunofluorescence assays were performed as previously described [89]. For the assay, primary antibodies were diluted 1:1000 for anti-HA mouse monoclonal antibody, 1:200 for anti-HA rabbit polyclonal antibody, and 1:10000 for anti-Igl mouse monoclonal antibody. To count filopodia, filopodia were visualized with an anti-Igl antibody, and the images of approximately 20 Z stacks of trophozoites, at 1-μm intervals, were collected using a Zeiss Meta510 confocal microscope; the clearest images, closest to the glass surface, were used for counting. The lengths and widths of filopodia were measured using ImageJ software (http://rsb.info.nih.gov/ij/), and filopodia longer than 0.7 μm in length, and narrower than 0.2 μm in width, were counted. The results were expressed as means ± standard deviations of three independent experiments in which 20 cells were examined.

### Measurement of adhesion to human erythrocytes

*E*. *histolytica* adhesion to human erythrocytes (HRBCs) (obtained from healthy volunteers in NIID) was measured as previously described [90]. Briefly, harvested trophozoites were washed and concentrated by resuspension in cold Opti-MEM (Life Technologies, MD, USA), and were then centrifuged at 300 g for 5 min at 4°C. HRBCs were obtained from healthy volunteers, of known ABO types, after providing written informed consent. HRBCs were washed and concentrated by resuspension in cold Opti-MEM twice, followed by centrifugation at 200 g for 5 min at 4°C. Approximately 10^5^ amebae and 5×10^6^ HRBCs were mixed in 1 mL Opti-MEM in a 1.5-ml tube; then, the resulting suspension was centrifuged at 200 g for 5 min at 4°C. After centrifugation, 0.9 mL of the supernatant was discarded, and the pellet was incubated on ice for 30 min. Following incubation, the pellet was resuspended in the remaining Opti-MEM by tapping the tube. A drop of the cell suspension was placed on a glass slide and examined under a microscope at 20x magnification. Fifteen images, per each strain, were captured in triplicated experiments. The number of HRBCs, attached to individual trophozoites, was counted for approximately 2,000 trophozoites for each experiment.

### Measurement of motility

Amoebic motility was measured as previously described [29]. Briefly, trophozoites were labeled using CellTracker Green CMFDA (Life Technologies, MD, USA), seeded on a 35-mm collagen-coated glass bottom culture dish (MatTek, MA, USA), incubated for 1 h, and examined at 35.50°C, under anaerobic conditions, using a Zeiss Meta510 confocal microscope with a 488-nm laser. Time-lapse images were collected every 5 s for 10 min and processed using ImageJ software. To quantify cell motility, cell migration was analyzed with open-source ICY software (http://icy.bioimageanalysis.org) using a tracking tool [91] that allows individual cell tracking throughout the time-lapse images. Average speeds were measured in 135 cells for EHI_176590-HA, and 106 cells for mock transformants, in four independent experiments.

### Immunoprecipitation and mass spectrometric analysis of EHI_176590 binding proteins

Immunoprecipitation and mass spectrometric analysis were conducted as previously described [92]. The quantitative value (QV), normalized with unweighted spectrum counts, was used to estimate the relative abundance of proteins in the samples. The proteins with a QV of >2.5, in the EHI_176590-HA sample, were selected as possible binding proteins and are listed in S3 Table.

### Experimental amoebic liver abscesses in hamsters

Approximately 3×10^6^ or 1×10^6^ trophozoites, from the ameba lines transfected with pEHI_176590-HA, pEhEx-04trig-EHI_176590, and the corresponding vector controls pEhEx-HA and pEhEx-04trig, were resuspended in 100 μL BI-S-33 medium and injected into the left lobe of the liver of 4-week-old female Syrian hamsters. The injected animals were sacrificed 6 days post-infection, and the livers and abscesses were dissected and weighed separately. All animal care procedures were in accordance with Standards Relating to the Care and Management of Laboratory Animals and Relief of Pain formulated by Ministry of the Environment.

### Statistical analysis

All experiments were performed in duplicate or triplicate and were repeated at least three times. The means and standard deviations were calculated, and statistical significance was determined by Student’s t test. A p value of <0.05 was considered statistically significant, unless otherwise noted.

### Transcriptome analysis

Transcriptome analysis was performed as previously described [25–27, 93]. Briefly, strains KU27 and KU50 were axenically grown in 25 cm^**2**^ tissue culture flasks; trophozoites were collected during the logarithmic growth phase in three independent experiments. Total RNA was extracted with TRIzol Reagent (Life Technologies, MD, USA) according to the manufacturer’s instructions. The concentration and quality of the RNA samples were analyzed with Nanodrop Spectrophotometer 1000 (Thermo Scientific, DE, USA) and Bio-Rad Experion Automated Electrophoresis System with RNA StdSens analysis kit, respectively. The first and second strand of cDNA were synthesized from 5 μg of total RNA using GeneChip One-Cycle cDNA Synthesis Kit (Affymetrix, CA, USA). Biotin-labeled cRNA was prepared using GeneChip IVT Labeling Kit (Affymetrix, CA, USA) and purified with GeneChip Sample Cleanup Module (Affymetrix, CA, USA) according to the manufacturers’ instructions. Prepared cRNA was fragmented and hybridized onto a custom-generated probe array chip (Eh_Eia520620F; Affymetrix, CA, USA). After hybridization, the arrays were washed and stained with streptavidin-phycoerythrin (Molecular Probes, OR, USA) using an Affymetrix GeneChip Fluidics Station 450. The arrays were scanned with an Affymetrix GeneChip Scanner 3000 at 570 nm. Data were analyzed with GeneSpring software by Tohoku-Kagaku-Yakuhin (Iwate, Japan). The relative level of mRNA was expressed after normalization using RNA polymerase II (EHI_056690). We confirmed the relatively stable expression of RNA polymerase II during oxidative stress compared to that of actin and glycerol 3-phosphate dehydrogenase genes ([93] and S8 Fig).

### Accession numbers

The genome sequences, reported here, were deposited under accession numbers DRA004163 (raw reads of the whole genomes of KU50 and KU27) and LC099939-41 (for HM-1:IMSS cl6, KU50, and KU27, respectively) for the region containing the *AIG1* gene described in this manuscript.

## Supporting information

S1 Table Statistics of HiSeq short reads and mapping status of *E*. *histolytica* KU27 and KU50 strains. (XLSX)

S2 Table Predicted high copy number genes and missing genes in *E*. *histolytica* KU27 and KU50. (XLSX)

S3 Table EHI_176540-HA binding proteins identified by affinity purification. (XLSX)

S4 Table Grouping and mutual inter-gene percentage identity of AIG1 family protein genes of *E*. *histolytica* HM-1:IMSS strain. (XLSX)

S5 Table RPKM comparisons of ORFs among KU27, KU50, and strains analyzed by Weedall et al. [20]. (XLSX)

S6 Table AIG1 family protein genes that were differently regulated by environmental changes in Weber et al. [13]. (XLSX)

S7 Table Amino acid substitutions characteristic of individual AIG1 family protein clusters. (XLSX)

S1 Fig The relative mRNA levels of the AIG1 family protein genes that are differentially present in KU27 and KU50. The transcript levels of the genes EHI_025990, EHI_039720, EHI_089440, and EHI_176590, in KU27 and KU50, were measured by DNA microarray and are shown as the percentage relative to that of the RNA polymerase II gene (EHI_056690). Gene IDs with “_at” indicate specific probe sets, and those with “_x_at” indicate that the probe set can detect more than one gene.
(TIF)

S2 Fig Validation of gene silencing of EHI_176590, and specificity of the anti- EHI_176590 antibody. (A) Validation of gene silencing of EHI_176590. Reverse transcriptase PCR, of the genes EHI_176590 and RNA polymerase II (EHI_056690), was performed using RNA from an EHI_176590 gene-silenced (gs) strain and pSAP2 mock vector transfected G3 strain. (B) Immuno-detection of EHI_176590 in EHI_176590 gene silenced and pSAP2 mock vector transfected G3 strains. Total cell lysates were analyzed by immunoblot analysis with an anti-EHI_176590 antibody. The arrow indicates the 32-kDa EHI_176590 protein, and asterisks indicate cross-reactive proteins.
(TIF)

S3 Fig Immunofluorescence assay of EHI_176590-HA without permeabilization. EHI_176590-HA-expressing cells were fixed, but not permeabilized, with detergents and were then reacted with an anti-HA antibody. Bar: 20 μm. Images obtained at low, intermediate, and high magnifications are shown.
(TIF)

S4 Fig Adhesion of EHI_176590-HA expressing, or mock, transformants to HRBCs. Microscopic images of trophozoites of EHI_176590-HA-expressing or mock (HA) transformants mixed with HRBCs. Bar: 100 μm.
(TIF)

S5 Fig Relative mRNA level of AIG1 family protein genes in HM1:IMSS, KU27, and KU50. Relative expression levels of indicated AIG1 family protein genes, normalized to the expression of RNA polymerase II gene, are shown. Probe sets labeled with “_at” represent a single gene, while “_s_at” or “_x_at” may recognize sequence form splice variants or other genes, respectively.
(TIF)

S6 Fig Adhesion of KU27 or KU50 to HRBCs. Microscopic images of trophozoites of KU27, or those of KU50, mixed with HRBCs. Bar: 100 μm (upper panels). Adhesion of KU27 and KU50 to HRBCs. KU27 (open bars) and KU50 (black bar) were co-cultured with HRBCs on ice for 30 min, and adherent HRBCs per ameba were counted. The total number of trophozoites was set to 100%, and the percentage of trophozoites, bound to 0–5, 6–10, or >10 HRBCs, are shown. Error bars indicate standard deviations for four biological replicates. *p-value <0.001.
(TIF)

S7 Fig Core genome phylogenetic analysis of *E*. *histolytica* strains, and volcano plot based on RPKM value of each ORF between KU27 and KU50. (A) Core genome phylogenetic analysis of *E*. *histolytica* strains. Genomes of indicated strains, except for KU27 and KU50, were obtained from Amoeba DB (http://amoebadb.org/amoeba/). Red and blue colors indicate virulent and avirulent strains, respectively. Single nucleotide variation (SNV), excluding biallele sites, was determined using VarScan v2.3.4 software. A phylogenetic tree was constructed based on 6,811 core genome SNVs, in *E*. *histolytica*, using the maximum-likelihood method with 1,000-fold bootstrapping. The numbers of pairwise SNVs are also shown in table format. Yellow boxes indicate SNVs between KU27 and KU50 (this study), and HM-1:IMSS and Rahman. (B) Volcano plot based on RPKM value of each ORF between KU27 and KU50. Statistical analysis of RPKM values, between KU27 and KU50, was performed using edgeR version 3.12.0 of the Bioconductor package [94]. The x-axis is log2 ratio of RPKM value (i.e., the putative gene copy number) between KU27 and KU50; the y-axis is adjusted p-value based on —log10. Dot lines on x- and y-axis show threshold of fold change (32-fold change) and p-value (p = 0.0001), respectively; light yellow boxes represent statistically significant areas. One red and two blue dots represent putative missing genes in KU27 and KU50, respectively. The AIG1 family protein of EHI_176590, which is a missing gene in KU27, is not shown in the volcano plot because of division by zero.
(TIF)

S8 Fig Expression level of control genes, and relative expression levels of EHI_176590, after normalization against three control genes. (A) Expression of RNA polymerase II, actin, and glycerol 3-phosphate dehydrogenase, in Cl6, are shown. Data are based on a previously published DNA microarray study [93]. (B) Relative mRNA levels of EHI_176590 AIG1 gene, during L-cysteine deprivation for 48 h [93], are shown after normalization against RNA polymerase II, actin, or G3PDH.
(TIF)

S9 Fig Features of AIG1 family proteins in *E*. *histolytica*. (A) Multiple sequence alignment (MSA) of the region around switch II of 28 AIG family proteins in *E*. *histolytica*. Entire sequences of the 28 proteins were aligned using MAFFT (v7.351b) [82], and visualized using MSAViewer [83]. Only the portion of MSA, which corresponds to the region around switch II, is shown. Predicted secondary structures of EHI_176590 are shown on the bottom. The three clusters are separated by dotted lines. Compared with the proteins of cluster 2 and 3, switch II of cluster 1 members tends to be diverged and elongated, except for EHI_176580. (B) Superposition of predicted 3D-models of the AIG1 domains in EHI_176590 (Cluster 1; cyan), EHI_129470 (Cluster 2; magenta), and EHI_022500 (Cluster 3; yellow). The models were constructed with Modeller (9v8) [95], based on alignments between the query, GIMAPs (PDB ID codes 2XTP (GIMAP2), and 3ZJC (GIMAP7), using FORTE [31]. Entire folds of the AIG1 domains, in these three proteins, are conserved, although EHI_176590 has a longer loop region in switch II (arrow). P-loop (red), switch II, and α2 are indicated in the figure. EHI_129470 and EHI_022500 correspond to the “center” sequences in each cluster, in terms of sequence identities. (C-F) MSA of 28 AIG family proteins in *E*. *histolytica*. Entire sequences of the 28 proteins were aligned using MAFFT (v7.351b) [82], and visualized using NCBI Multiple Sequence Alignment Viewer 1.6.0. Predicted secondary structures of EHI_176590 are shown on the bottom. The three clusters are separated by black dotted lines and the region differences between clusters are indicated by the red dotted square. Different regions, within the AIG1 domain, are also indicated in predicted 3D-model (lower panel in C-E). The differences between the three clusters are summarized in S7 Table. The proximal location of C138 and C181, located at a distinct position in the MSA of 28 sequences (see S9D and S9E Fig), may imply the formation of a disulfide bond in EHI_176590.
(TIF)

S10 Fig Phallacidin staining of the transformant expressing HA-tagged EHI_176590. Trophozoites were fixed with 3.7% paraformaldehyde, permeabilized with 0.2% saponin, incubated with 0.33 μM of BODIPY FL phallacidin (Molecular Probes, Eugine, OR) for 20 min, and stained with anti-HA antibody followed by anti-mouse Alexa 568 conjugated secondary antibody. The samples were examined on a Carl-Zeiss LSM 510 META confocal laser-scanning microscope, and images were analyzed using LSM510 software. Note that phallacidin staining is not concentrated in the protrusions.
(TIF)
