# AIG1 affects in vitro and in vivo virulence in clinical isolates of *Entamoeba histolytica*

**DOI:** 10.1371/journal.ppat.1007091

**Published:** 2018-05-31

**Authors:** Kumiko Nakada-Tsukui, Tsuyoshi Sekizuka, Emi Sato-Ebine, Aleyla Escueta-de Cadiz, Dar-der Ji, Kentaro Tomii, Makoto Kuroda, Tomoyoshi Nozaki

There is an error in the Acknowledgements section: Prof. Yuji Tachibana should be Dr. Hiroshi Tachibana. The correct Acknowledgements section should read: We thank Dr. Seiki Kobayashi of the Keio University School of Medicine, and Dr. Hiroshi Tachibana of the Tokai University School of Medicine, for providing E. histolytica clinical strains and nucleotide samples of clinical samples. We thank Dr. Nicholas E. Sherman, W.M. Keck Biomedical Mass Spectrometry Laboratory, University of Virginia for assistance with mass spectrometric analysis. We thank Ms. Kumiko Shibata and Mihoko Imada for technical assistance.
