# Correction: Correction: AIG1 affects in vitro and in vivo virulence in clinical isolates of *Entamoeba histolytica*

**DOI:** 10.1371/journal.ppat.1007258

**Published:** 2018-08-17

**Authors:** The PLOS Pathogens Staff

There is an error in the title of the correction published on May 31, 2018. The correct title is: Correction: AIG1 affects in vitro and in vivo virulence in clinical isolates of *Entamoeba histolytica*. The correct citation is: Nakada-Tsukui K, Sekizuka T, Sato-Ebine E, Escueta-de Cadiz A, Ji D-d, Tomii K, et al. (2018) Correction: AIG1 affects in vitro and in vivo virulence in clinical isolates of *Entamoeba histolytica*. PLoS Pathog 14(5): e1007091.

The publisher apologizes for the error.
