# Supplementary figures and images for: AIG1 affects in vitro and in vivo virulence in clinical isolates of Entamoeba histolytica
Source: PLoS Pathog. 2018 Mar 19;14(3):e1006882. doi: 10.1371/journal.ppat.1006882 (PMC5884625; doi:10.1371/journal.ppat.1006882)

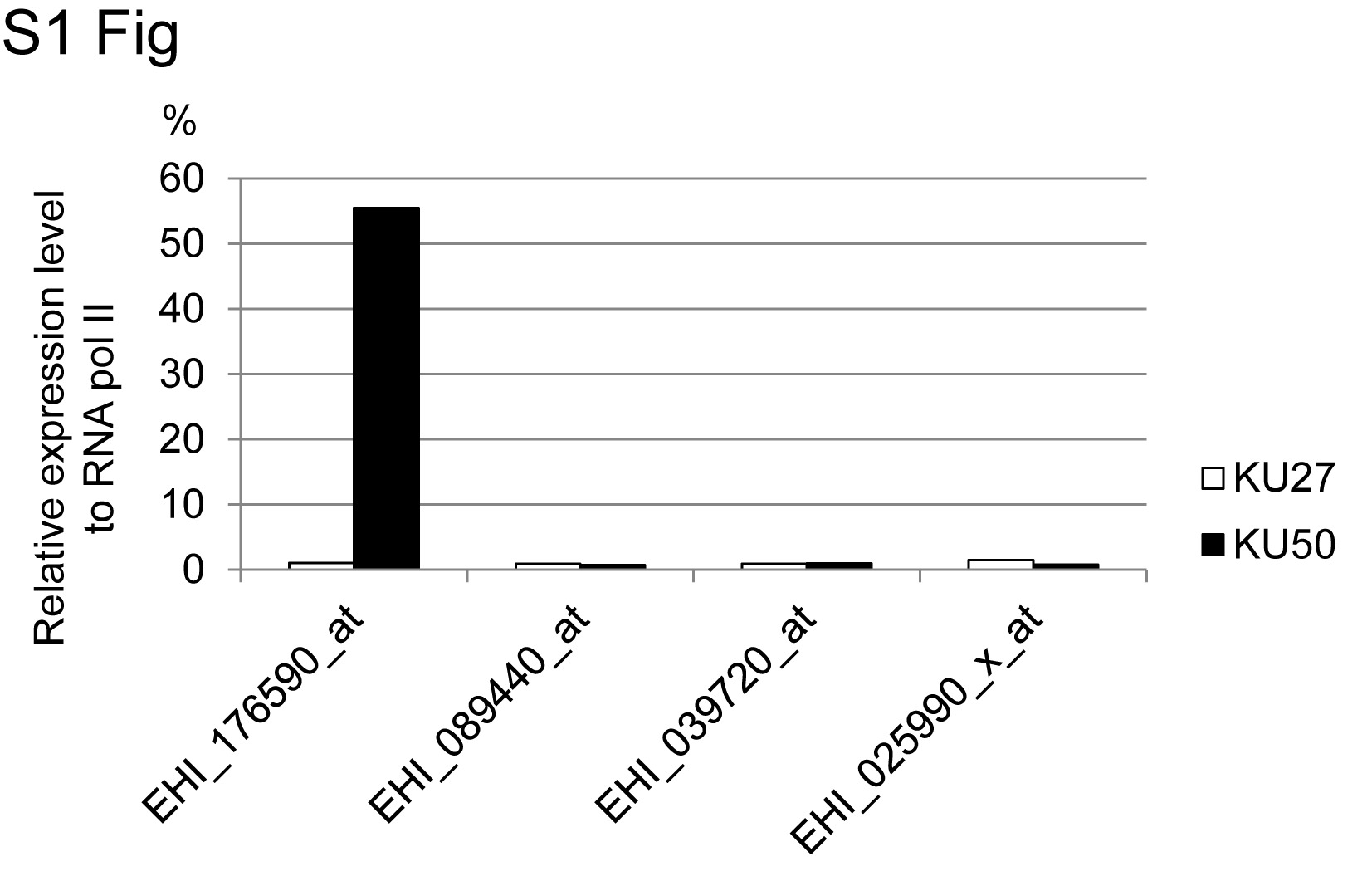

Supplement: S1 Fig — The transcript levels of the genes EHI_025990, EHI_039720, EHI_089440, and EHI_176590, in KU27 and KU50, were measured by DNA microarray and are shown as the percentage relative to that of the RNA polymerase II gene (EHI_056690). Gene IDs with “_at” indicate specific probe sets, and those with “_x_at” indicate that the probe set can detect more than one gene. (TIF) [file ppat.1006882.s008.tif]

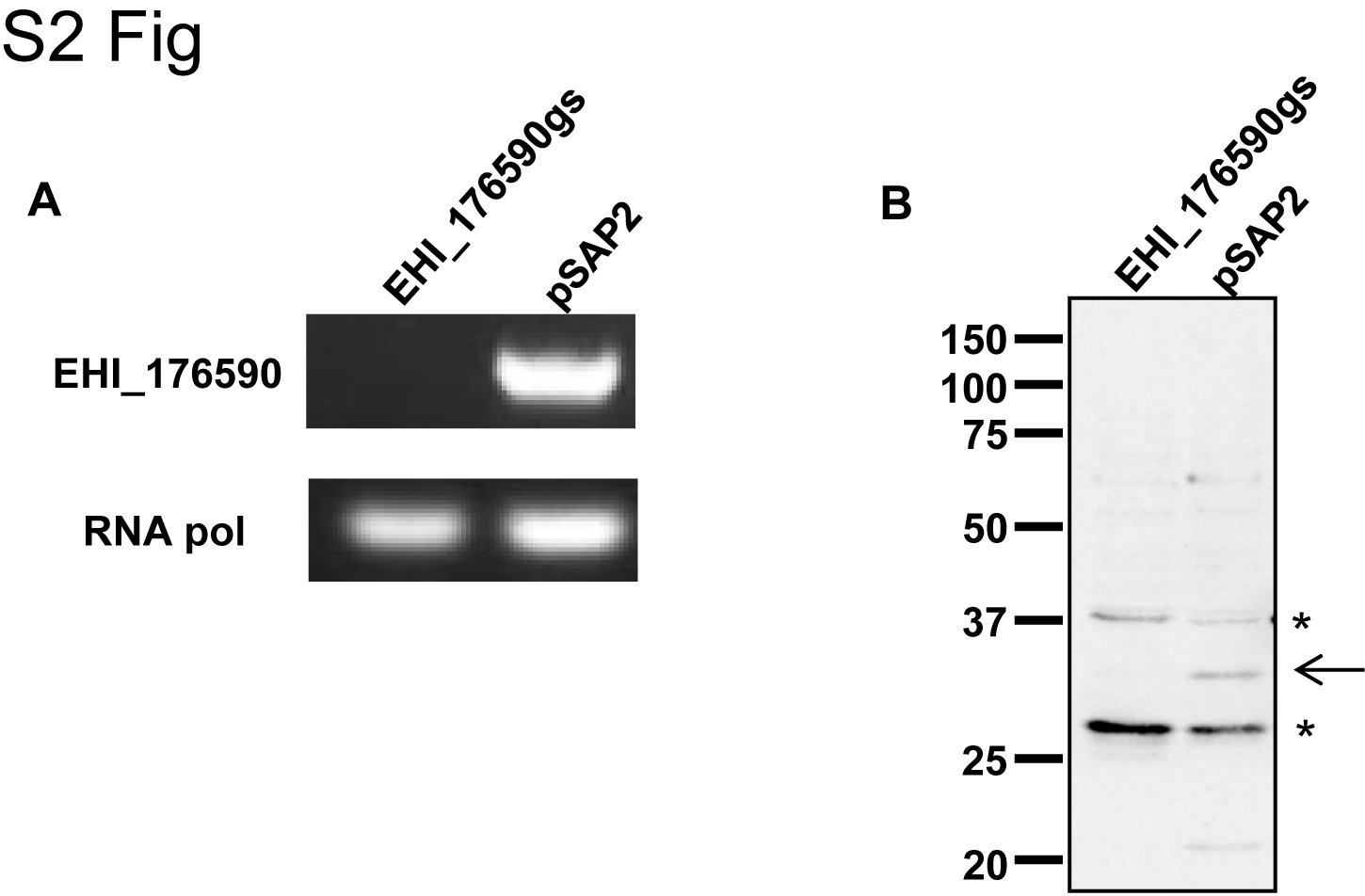

Supplement: S2 Fig — (A) Validation of gene silencing of EHI_176590. Reverse transcriptase PCR, of the genes EHI_176590 and RNA polymerase II (EHI_056690), was performed using RNA from an EHI_176590 gene-silenced (gs) strain and pSAP2 mock vector transfected G3 strain. (B) Immuno-detection of EHI_176590 in EHI_176590 gene silenced and pSAP2 mock vector transfected G3 strains. Total cell lysates were analyzed by immunoblot analysis with an anti-EHI_176590 antibody. The arrow indicates the 32-kDa EHI_176590 protein, and asterisks indicate cross-reactive proteins. (TIF) [file ppat.1006882.s009.tif]

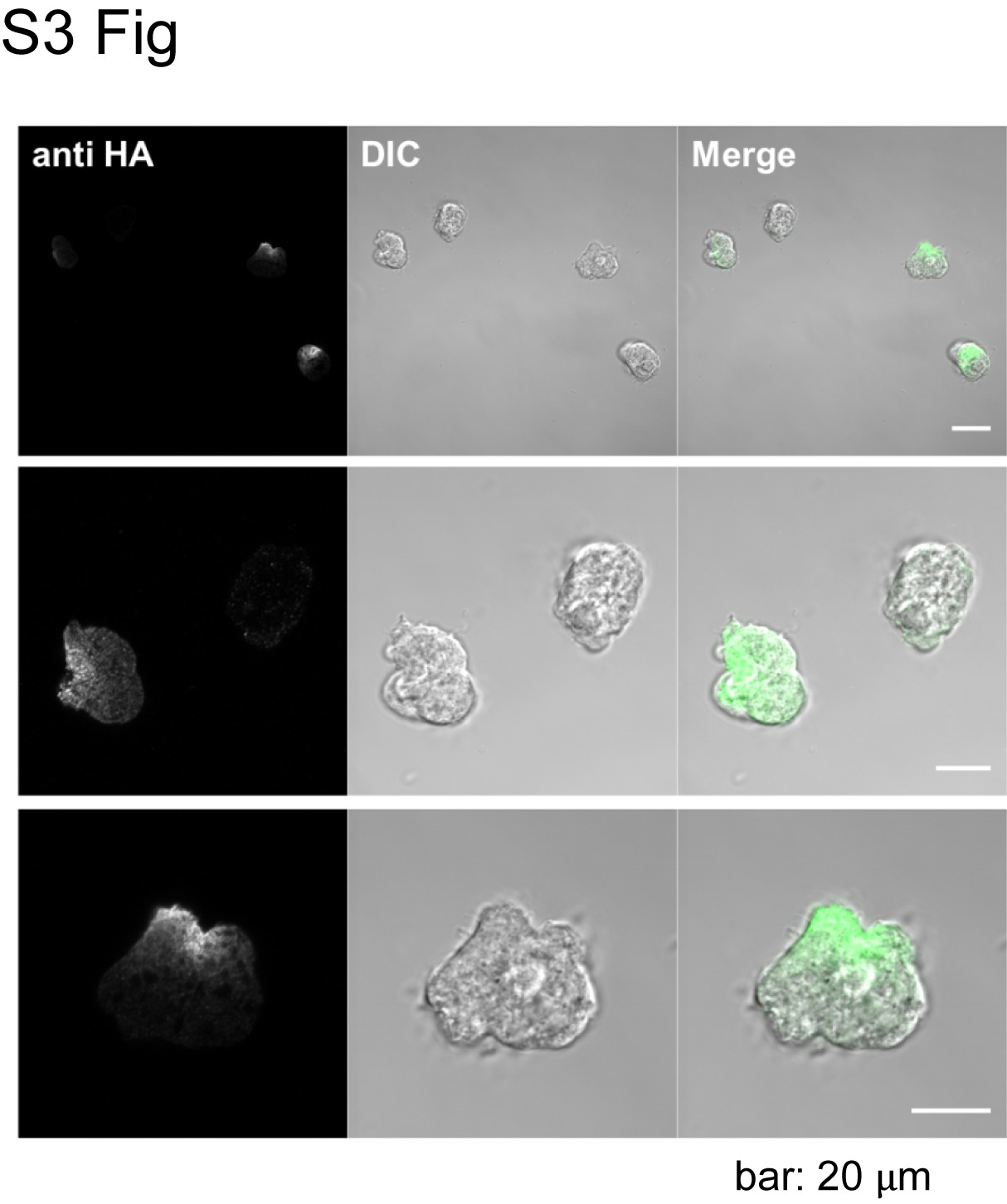

Supplement: S3 Fig — EHI_176590-HA-expressing cells were fixed, but not permeabilized, with detergents and were then reacted with an anti-HA antibody. Bar: 20 μm. Images obtained at low, intermediate, and high magnifications are shown. (TIF) [file ppat.1006882.s010.tif]

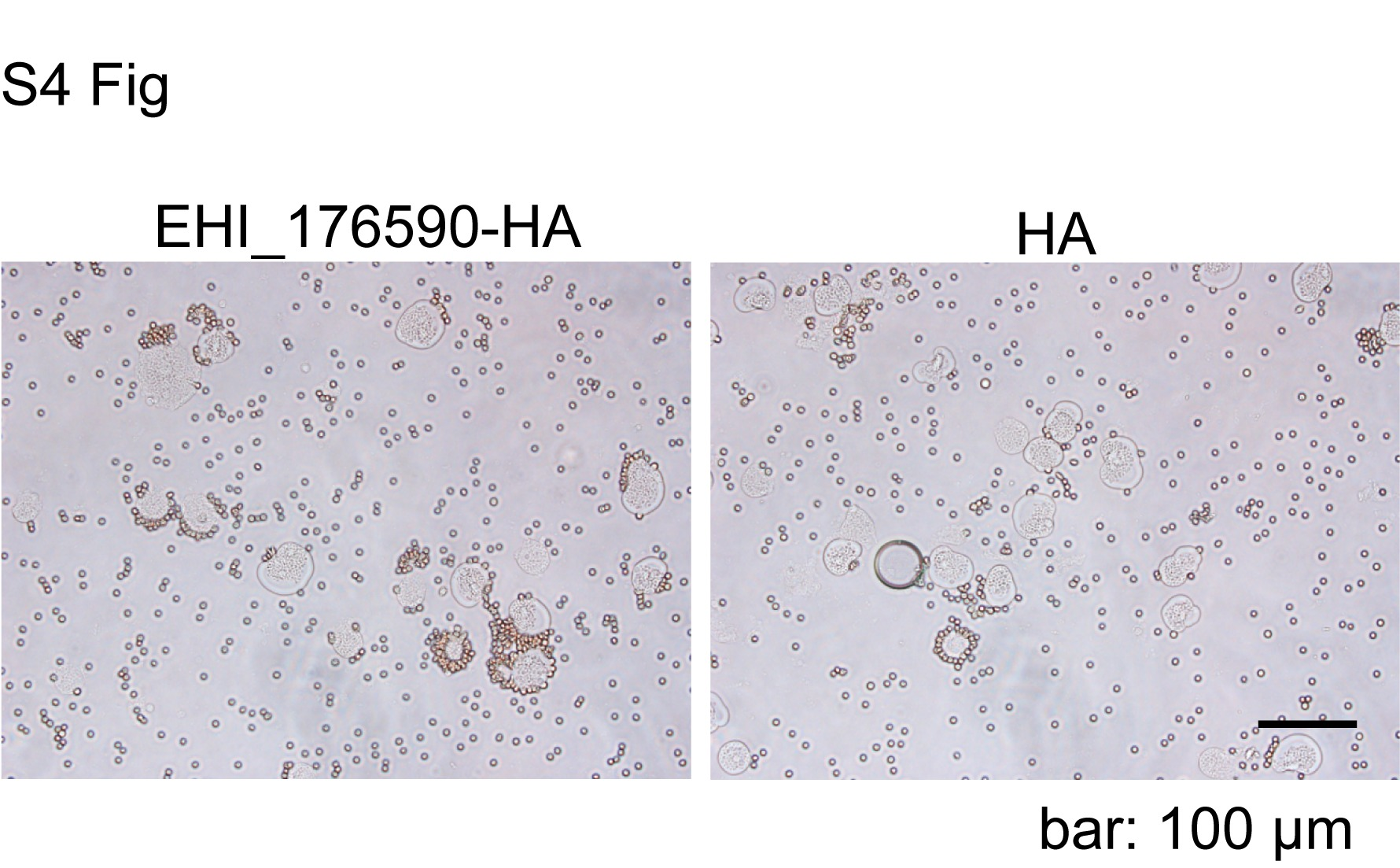

Supplement: S4 Fig — Microscopic images of trophozoites of EHI_176590-HA-expressing or mock (HA) transformants mixed with HRBCs. Bar: 100 μm. (TIF) [file ppat.1006882.s011.tif]

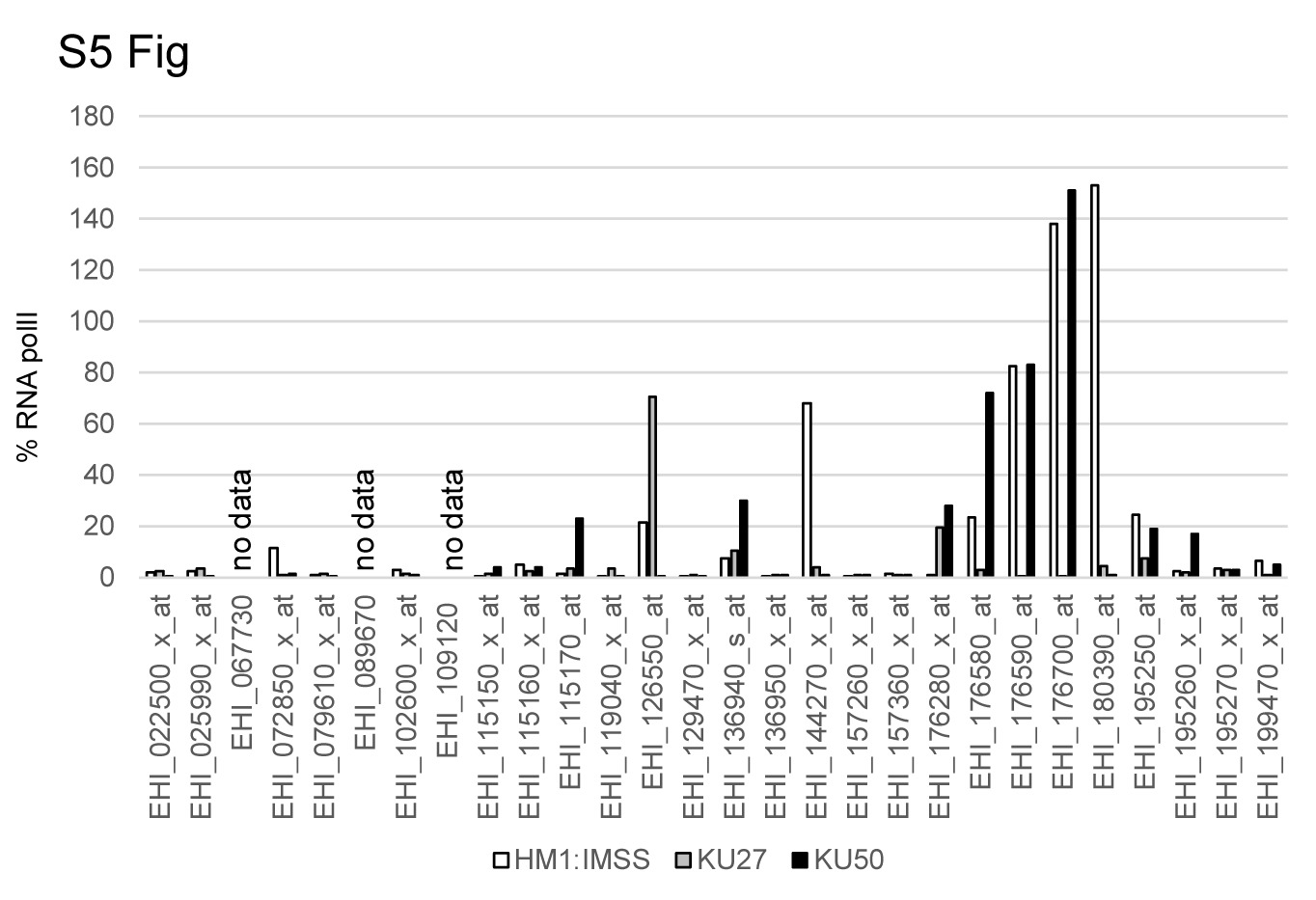

Supplement: S5 Fig — Relative expression levels of indicated AIG1 family protein genes, normalized to the expression of RNA polymerase II gene, are shown. Probe sets labeled with “_at” represent a single gene, while “_s_at” or “_x_at” may recognize sequence form splice variants or other genes, respectively. (TIF) [file ppat.1006882.s012.tif]

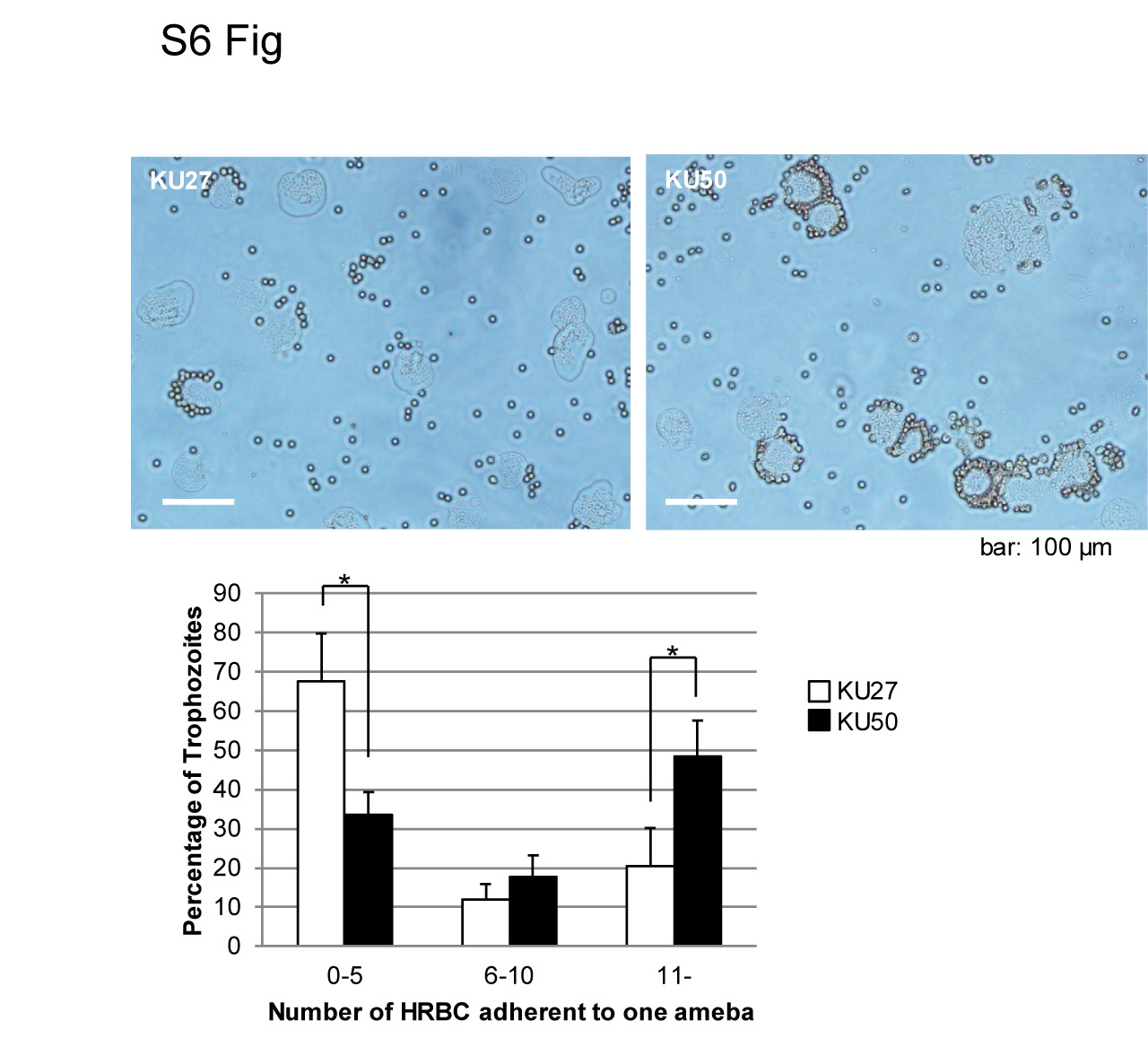

Supplement: S6 Fig — Microscopic images of trophozoites of KU27, or those of KU50, mixed with HRBCs. Bar: 100 μm (upper panels). Adhesion of KU27 and KU50 to HRBCs. KU27 (open bars) and KU50 (black bar) were co-cultured with HRBCs on ice for 30 min, and adherent HRBCs per ameba were counted. The total number of trophozoites was set to 100%, and the percentage of trophozoites, bound to 0–5, 6–10, or >10 HRBCs, are shown. Error bars indicate standard deviations for four biological replicates. *p-value <0.001. (TIF) [file ppat.1006882.s013.tif]

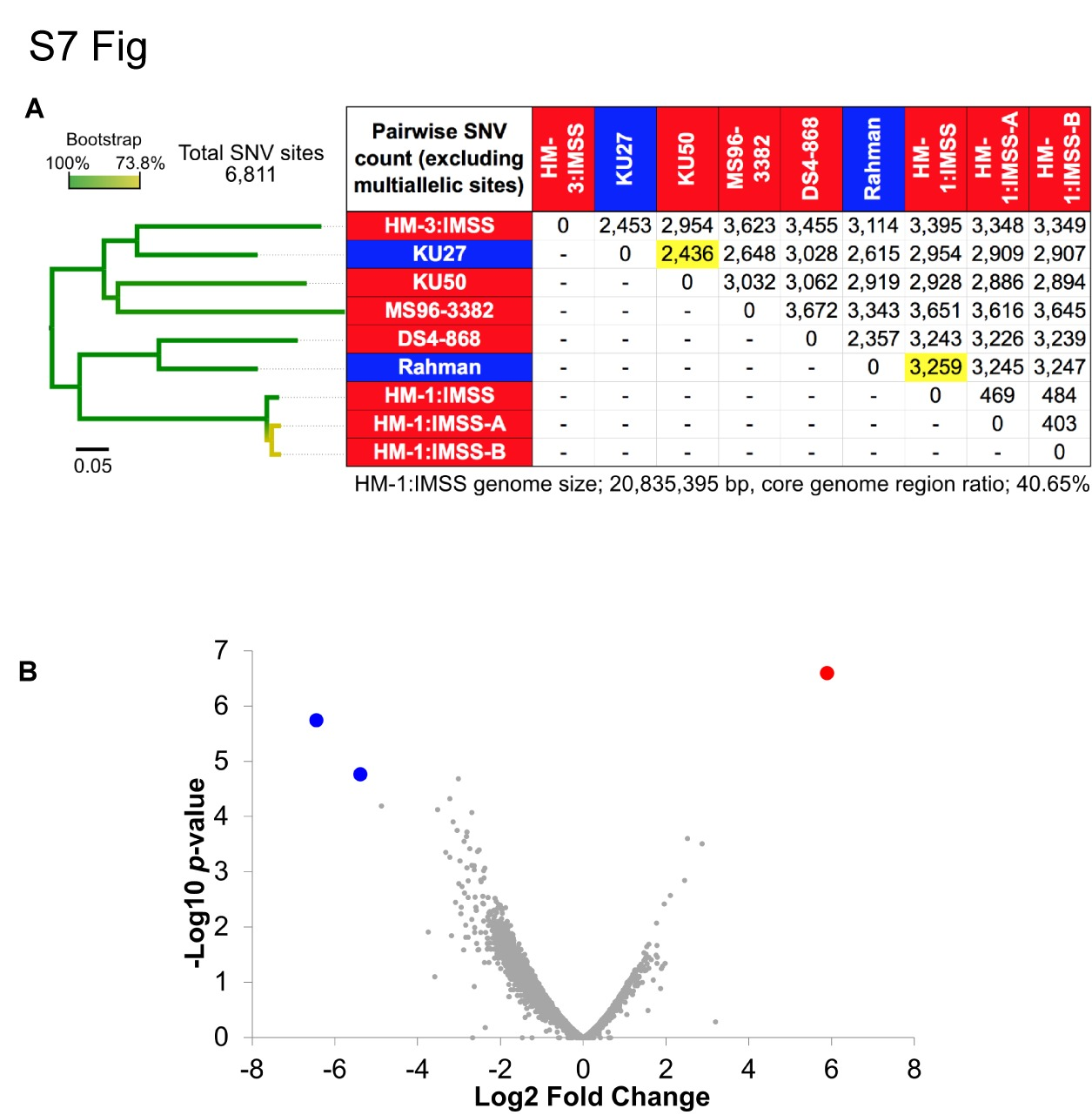

Supplement: S7 Fig — (A) Core genome phylogenetic analysis of E. histolytica strains. Genomes of indicated strains, except for KU27 and KU50, were obtained from Amoeba DB (http://amoebadb.org/amoeba/). Red and blue colors indicate virulent and avirulent strains, respectively. Single nucleotide variation (SNV), excluding biallele sites, was determined using VarScan v2.3.4 software. A phylogenetic tree was constructed based on 6,811 core genome SNVs, in E. histolytica, using the maximum-likelihood method with 1,000-fold bootstrapping. The numbers of pairwise SNVs are also shown in table format. Yellow boxes indicate SNVs between KU27 and KU50 (this study), and HM-1:IMSS and Rahman. (B) Volcano plot based on RPKM value of each ORF between KU27 and KU50. Statistical analysis of RPKM values, between KU27 and KU50, was performed using edgeR version 3.12.0 of the Bioconductor package [94]. The x-axis is log2 ratio of RPKM value (i.e., the putative gene copy number) between KU27 and KU50; the y-axis is adjusted p-value based on —log10. Dot lines on x- and y-axis show threshold of fold change (32-fold change) and p-value (p = 0.0001), respectively; light yellow boxes represent statistically significant areas. One red and two blue dots represent putative missing genes in KU27 and KU50, respectively. The AIG1 family protein of EHI_176590, which is a missing gene in KU27, is not shown in the volcano plot because of division by zero. (TIF) [file ppat.1006882.s014.tif]

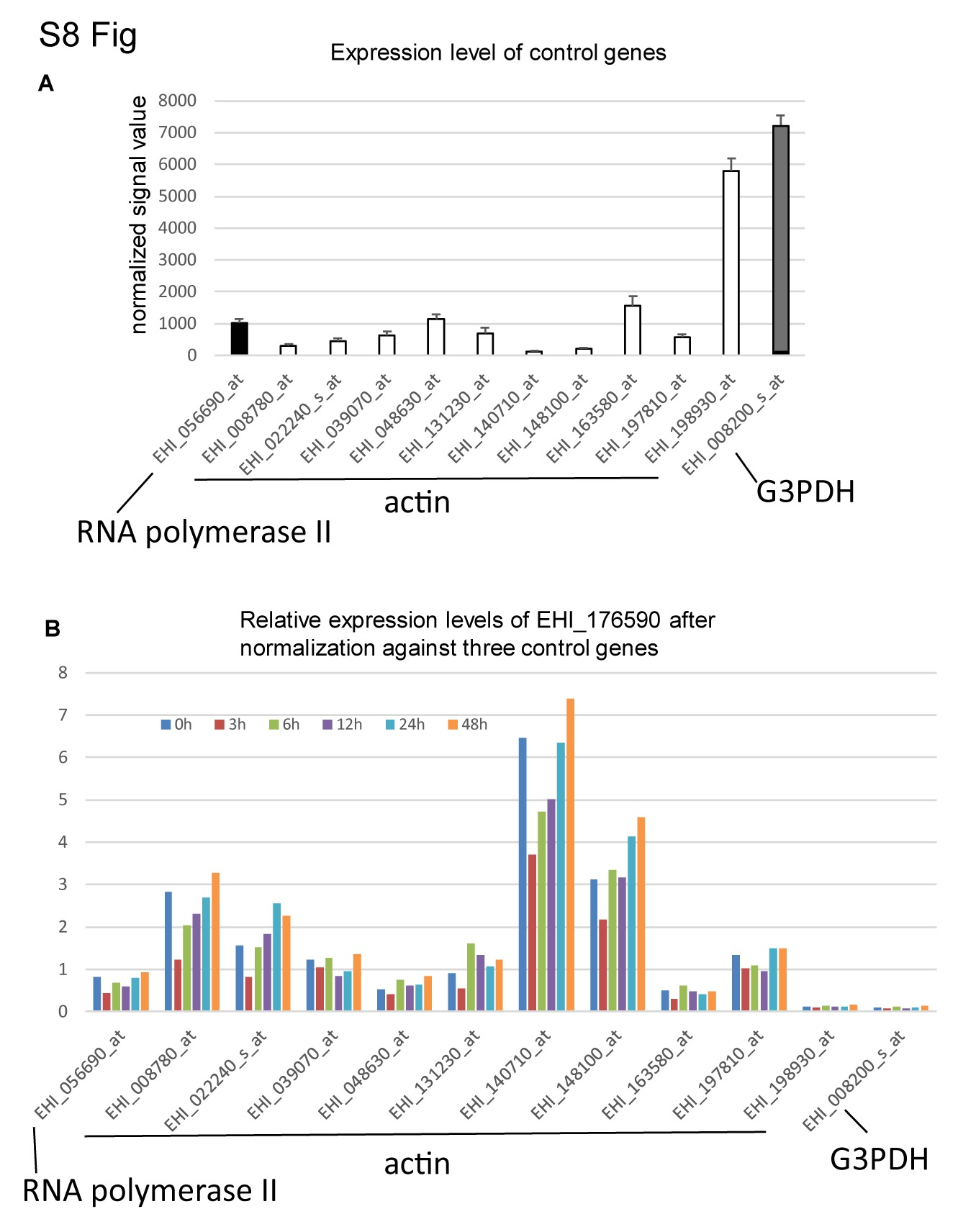

Supplement: S8 Fig — (A) Expression of RNA polymerase II, actin, and glycerol 3-phosphate dehydrogenase, in Cl6, are shown. Data are based on a previously published DNA microarray study [93]. (B) Relative mRNA levels of EHI_176590 AIG1 gene, during L-cysteine deprivation for 48 h [93], are shown after normalization against RNA polymerase II, actin, or G3PDH. (TIF) [file ppat.1006882.s015.tif]

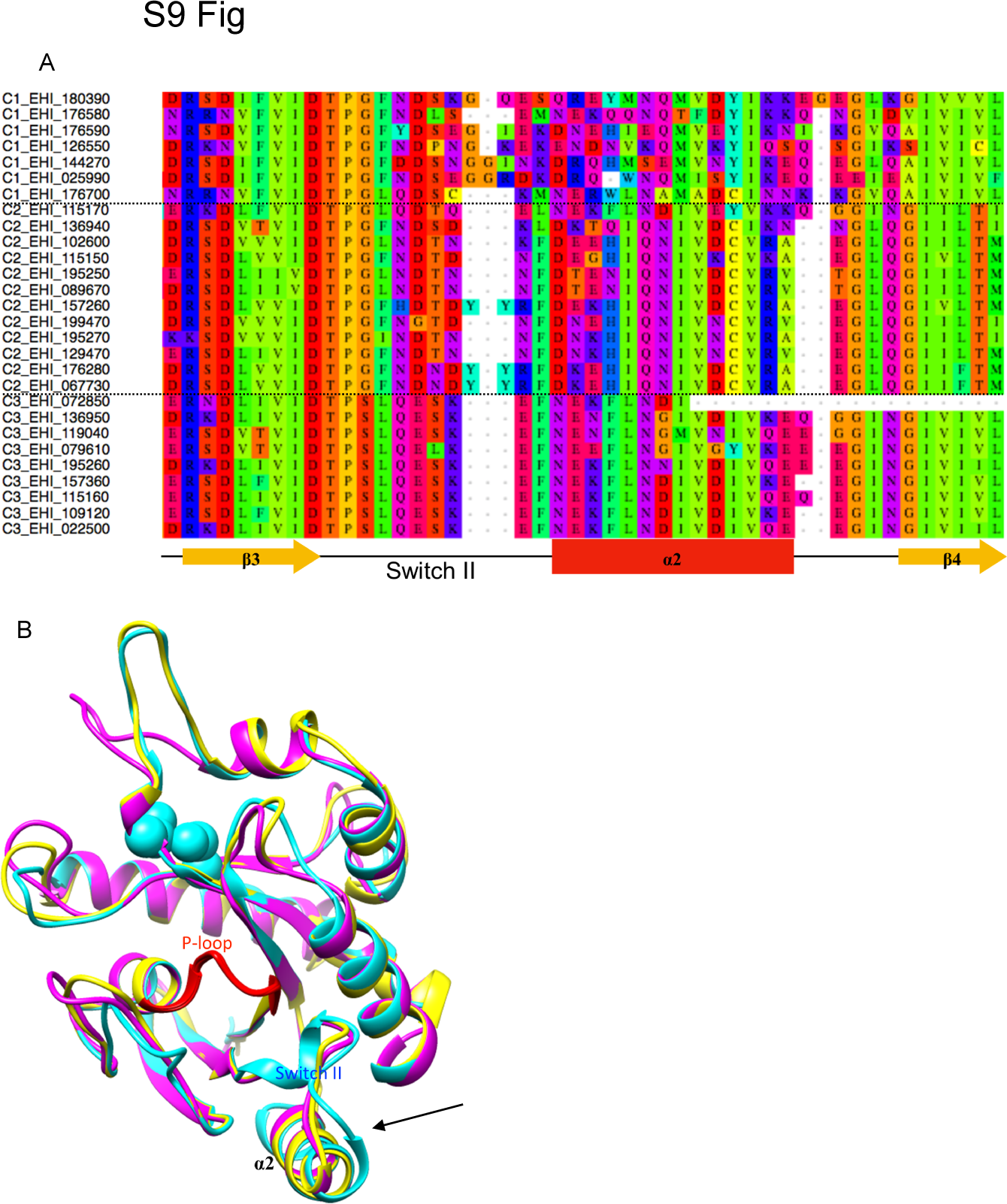

Supplement: S9 Fig — (A) Multiple sequence alignment (MSA) of the region around switch II of 28 AIG family proteins in E. histolytica. Entire sequences of the 28 proteins were aligned using MAFFT (v7.351b) [82], and visualized using MSAViewer [83]. Only the portion of MSA, which corresponds to the region around switch II, is shown. Predicted secondary structures of EHI_176590 are shown on the bottom. The three clusters are separated by dotted lines. Compared with the proteins of cluster 2 and 3, switch II of cluster 1 members tends to be diverged and elongated, except for EHI_176580. (B) Superposition of predicted 3D-models of the AIG1 domains in EHI_176590 (Cluster 1; cyan), EHI_129470 (Cluster 2; magenta), and EHI_022500 (Cluster 3; yellow). The models were constructed with Modeller (9v8) [95], based on alignments between the query, GIMAPs (PDB ID codes 2XTP (GIMAP2), and 3ZJC (GIMAP7), using FORTE [31]. Entire folds of the AIG1 domains, in these three proteins, are conserved, although EHI_176590 has a longer loop region in switch II (arrow). P-loop (red), switch II, and α2 are indicated in the figure. EHI_129470 and EHI_022500 correspond to the “center” sequences in each cluster, in terms of sequence identities. (C-F) MSA of 28 AIG family proteins in E. histolytica. Entire sequences of the 28 proteins were aligned using MAFFT (v7.351b) [82], and visualized using NCBI Multiple Sequence Alignment Viewer 1.6.0. Predicted secondary structures of EHI_176590 are shown on the bottom. The three clusters are separated by black dotted lines and the region differences between clusters are indicated by the red dotted square. Different regions, within the AIG1 domain, are also indicated in predicted 3D-model (lower panel in C-E). The differences between the three clusters are summarized in S7 Table. The proximal location of C138 and C181, located at a distinct position in the MSA of 28 sequences (see S9D and S9E Fig), may imply the formation of a disulfide bond in EHI_176590. (TIF) [file ppat.1006882.s016.tif]

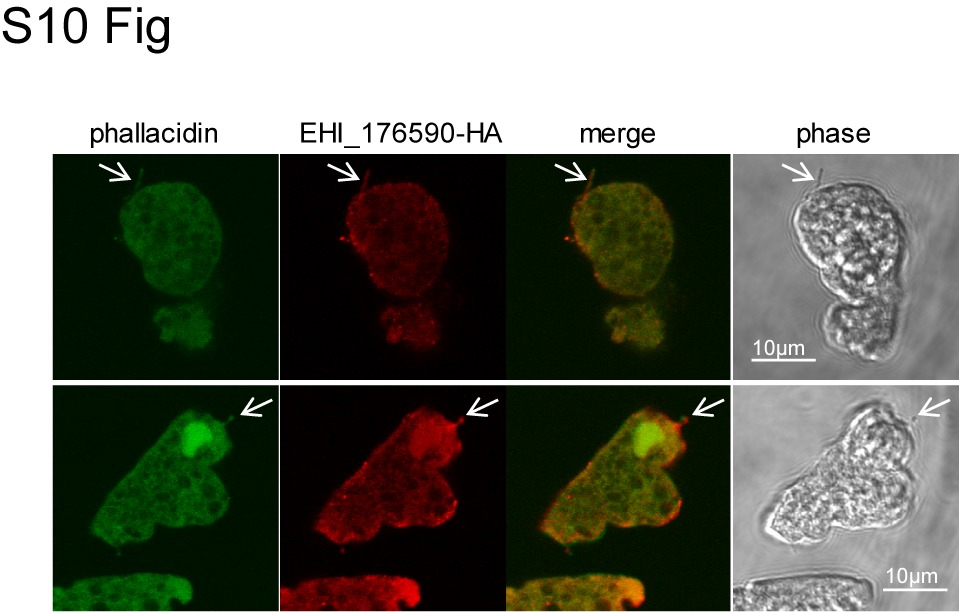

Supplement: S10 Fig — Trophozoites were fixed with 3.7% paraformaldehyde, permeabilized with 0.2% saponin, incubated with 0.33 μM of BODIPY FL phallacidin (Molecular Probes, Eugine, OR) for 20 min, and stained with anti-HA antibody followed by anti-mouse Alexa 568 conjugated secondary antibody. The samples were examined on a Carl-Zeiss LSM 510 META confocal laser-scanning microscope, and images were analyzed using LSM510 software. Note that phallacidin staining is not concentrated in the protrusions. (TIF) [file ppat.1006882.s017.tif]
